# Structure and metabolic potential of the prokaryotic communities from the hydrothermal system of Paleochori Bay, Milos, Greece

**DOI:** 10.3389/fmicb.2022.1060168

**Published:** 2023-01-06

**Authors:** Sven Le Moine Bauer, Guang-Sin Lu, Steven Goulaouic, Valentine Puzenat, Anders Schouw, Thibaut Barreyre, Vera Pawlowsky-Glahn, Juan José Egozcue, Jean-Emmanuel Martelat, Javier Escartin, Jan P. Amend, Paraskevi Nomikou, Othonas Vlasopoulos, Paraskevi Polymenakou, Steffen Leth Jørgensen

**Affiliations:** ^1^Center for Deep Sea Research, Department of Earth Science, University of Bergen, Bergen, Norway; ^2^Cooperative Institute for Climate, Ocean and Ecosystem Studies, University of Washington, Seattle, WA, United States; ^3^NOAA Pacific Marine Environmental Laboratory, Seattle, WA, United States; ^4^Institut de Physique du Globe de Paris, CNRS, Université Paris Cité, Paris, France; ^5^Center for Deep Sea Research, Department of Biology, University of Bergen, Bergen, Norway; ^6^Department of Computer Science, Applied Mathematics and Statistics, University of Girona, Girona, Spain; ^7^Department of Civil and Environmental Engineering, University Politécnica de Cataluña, Barcelona, Spain; ^8^Université de Lyon, UCBL, ENSL, CNRS, Laboratoire de Géologie LGL-TPE, Villeurbanne, France; ^9^Laboratoire de Géologie (CNRS UMR8538), Ecole Normale Supérieure de Paris, PSL University, Paris, France; ^10^Departments of Earth Sciences and Biological Sciences, University of Southern California, Los Angeles, CA, United States; ^11^Faculty of Geology and Geoenvironment, National and Kapodistrian University of Athens, Athens, Greece; ^12^Institute of Marine Biology Biotechnology and Aquaculture, Hellenic Center for Marine Research, Heraklion, Greece

**Keywords:** Milos, shallow hydrothermal vent field, microbial community, functional genes, 16S rRNA sequencing, thermal gradient

## Abstract

**Introduction:**

Shallow hydrothermal systems share many characteristics with their deep-sea counterparts, but their accessibility facilitates their study. One of the most studied shallow hydrothermal vent fields lies at Paleochori Bay off the coast of Milos in the Aegean Sea (Greece). It has been studied through extensive mapping and its physical and chemical processes have been characterized over the past decades. However, a thorough description of the microbial communities inhabiting the bay is still missing.

**Methods:**

We present the first in-depth characterization of the prokaryotic communities of Paleochori Bay by sampling eight different seafloor types that are distributed along the entire gradient of hydrothermal influence. We used deep sequencing of the 16S rRNA marker gene and complemented the analysis with qPCR quantification of the 16S rRNA gene and several functional genes to gain insights into the metabolic potential of the communities.

**Results:**

We found that the microbiome of the bay is strongly influenced by the hydrothermal venting, with a succession of various groups dominating the sediments from the coldest to the warmest zones. Prokaryotic diversity and abundance decrease with increasing temperature, and thermophilic archaea overtake the community.

**Discussion:**

Relevant geochemical cycles of the Bay are discussed. This study expands our limited understanding of subsurface microbial communities in acidic shallow-sea hydrothermal systems and the contribution of their microbial activity to biogeochemical cycling.

## Introduction

Marine hydrothermalism is a phenomenon where seawater percolates though the crust or the sediments, becomes heated by volcanic or tectonic activity, and returns to the seafloor in the form of focused or diffuse hydrothermal venting. During its journey through the subsurface, the seawater reacts with the substrate, resulting in hydrothermal fluids that are often enriched in metals and reduced compounds while being depleted in oxidized compounds (Alt, 1995). At venting sites, the physical and chemical gradients generated by the mixing of the fluids and the seawater allows for the growth of unique microbial communities (Orcutt et al., 2011; Price and Giovannelli, 2017). While such hydrothermal vents can be found at all depths, a separation is made around 200 m depth to differentiate deep and dark hydrothermal systems on one hand and shallow and photic hydrothermal systems on the other hand (Tarasov et al., 2005; Price and Giovannelli, 2017).

As of 2020, around 70 shallow systems are reported in the InterRidge 3.4 database (Beaulieu and Szafranski, 2020). While they occur in a variety of tectonically active settings, they are mostly associated with submarine volcanism, island and intra-oceanic arcs, ridge environments, intraplate oceanic volcanism, continental margins, and rift basins (Tarasov et al., 2005) and can be found all around the world (see for a review Price and Giovannelli, 2017). Despite similarities with their deep-sea counterparts, shallow systems present also significant particularities, such as the presence of light allowing photosynthesis, and the common presence of a gas phase due to a lower hydrostatic pressure than in the deep sea. They are furthermore exposed to stronger time-dependent external forcing such as tidal influence, wind- and wave-driven circulation, and coastal processes such as land-derived nutrient loading (Chen et al., 2005; Yücel et al., 2013; Price et al., 2015; Price and Giovannelli, 2017).

**Figure 1:**
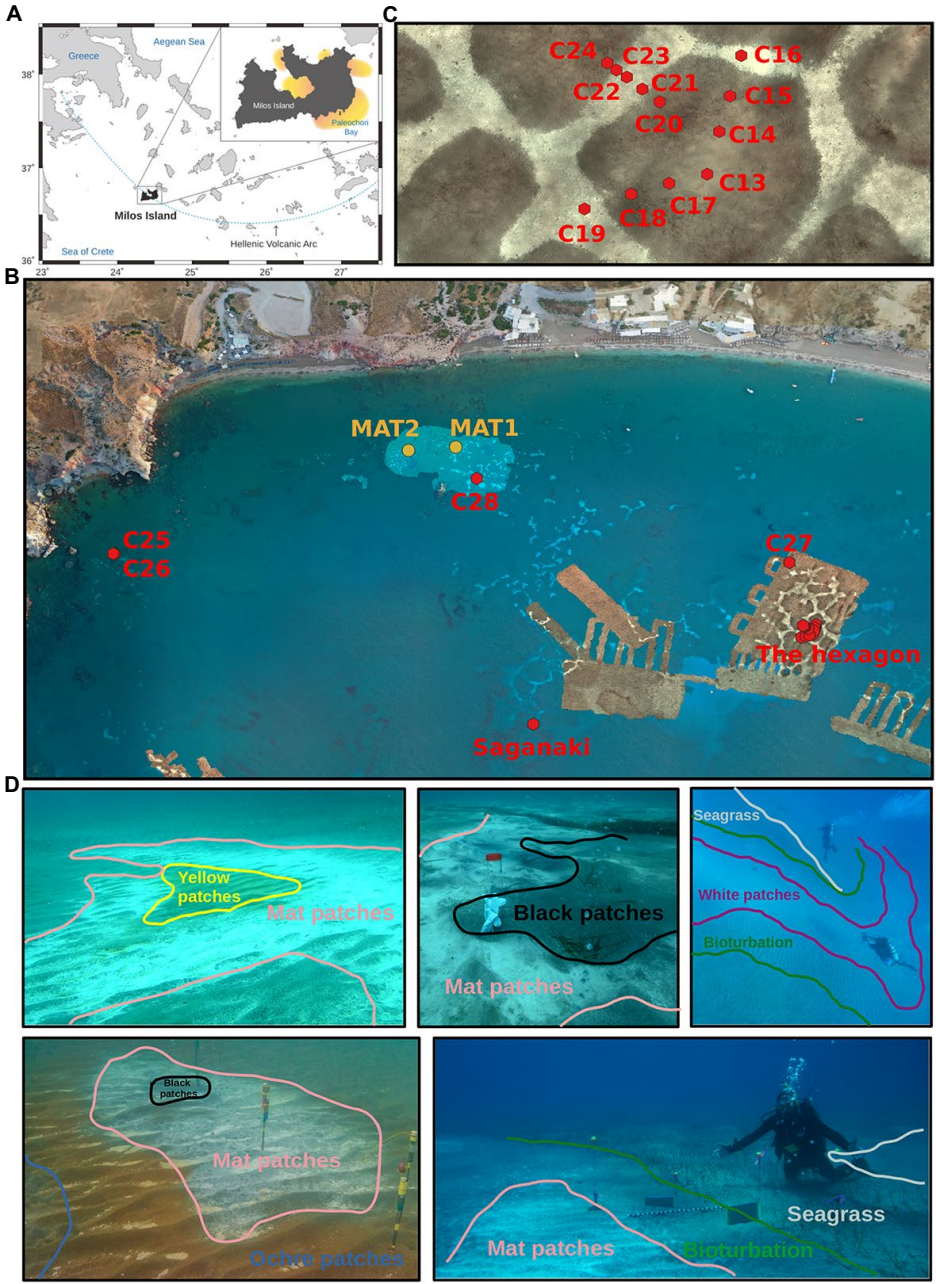
Map and photographies of the sampling locations. **(A)** Localization of Milos and Paleochori Bay. **(B)** Localization of the cores taken in Paleochori Bay. **(C)** Zoom into the location of “The hexagon.” **(D)** Photographies of the various types of seafloor investigated here. The lower right picture represents the Saganaki vent field. The colors used are consistent throughout the figures, with light blue/grey for seagrass patches, green for bioturbation, blue for ochre patches, dark pink for white patches, salmon for mat patches, black for black patches, and yellow for yellow patches. See Puzenat et al. (2021) for details on the publicly available AUV and drone background photomosaics in **(B,C)** (Martelat et al., 2019; Puzenat et al., 2019a,b).

Milos Island and its surroundings, situated on the Hellenic Volcanic Arc in the South Aegan Sea, is one of the most studied shallow hydrothermal vent system (Figure 1A). This arc was formed by the convergence of the African plate beneath the Aegean micro-plate and has been volcanically active since the Pliocene (Varnavas and Cronan, 2005; Jolivet et al., 2013). The subduction results in magmas of intermediate to felsic composition, influenced by andesitic to dacitic volcanic rocks and low-grade (greenschist facies) metamorphic rocks that compose the host rock at Milos (see Fytikas et al., 1986, and references within). Overlying the igneous and metamorphic rocks are carbonate-rich sediments with elevated concentrations of lead and zinc (Karageorgis et al., 1998). Although the last volcanic eruption at Milos was ~90 ka ago, remnant heat from the quiescent magma system still drives hydrothermal circulation on land and offshore, making it one of the largest shallow-sea hydrothermal systems described to date, covering ~35 km^2^ (Dando et al., 1995a). The most intense submarine venting identified to date occurs at Paleochori Bay (Figure 1A), off the south-eastern coastline at water depths from 3 m to at least 300 m (Dando et al., 2000). The venting fluids are chemically reduced, rich in sulfide and mercury, and acidic (pH ~ 4.4), with temperatures up to 122°C (Dando et al., 1995a, 2000; Valsami-Jones et al., 2005; Price R.E. et al., 2013; Roberts et al., 2021). Furthermore, the fluids from Milos have to date the highest arsenic concentrations of any submarine vent analyzed, with concentrations approximately 3,000 times higher than seawater values (Price et al., 2015). The gas phase is mainly composed of CO_2_ (commonly exceeding 90%), with lower amounts of H_2_, H_2_S and CH_4_ (Dando et al., 1995a). Low salinity fluids can also be found with different metal enrichments, resulting from the reliquefaction of the gas phase (Valsami-Jones et al., 2005). The hydrothermal discharge mixes with oxic, slightly alkaline seawater to produce white, yellow, orange, and brown manganese, iron, arsenic and sulfur mineral precipitates (Wenzhöfer et al., 2000; Kotopoulou et al., 2022) that are easily visible using satellite, drone, or underwater vehicle imagery (Khimasia et al., 2020; Martelat et al., 2020; Puzenat et al., 2021). This hydrothermalised seafloor provides surfaces and sources of nutrients and energy for microbial communities at or near the seafloor (Dando et al., 1998; Price R.E. et al., 2013; Gilhooly et al., 2014; Godelitsas et al., 2015).

Microbes in a system like Milos play several ecological roles, by fixing CO_2_ through chemo- and photolithoautotrophy, remineralizing organic matter, and mediating several metal cycles. Dando et al. (1995b) and Sievert et al. (1999) were the first to study the microbiology of the hydrothermal field of Paleochori Bay on Milos, followed by Brinkhoff et al. (1999) which focused on sulfur oxidizers. Since, several new microbial species have been isolated from the bay (Jochimsen et al., 1997; Arab et al., 2000; Sievert and Kuever, 2000; Schlesner et al., 2001), while other studies have looked at the microbial communities in general using most probable numbers, denaturing gradient gel electrophoresis, fluorescence *in situ* hybridization, lipid analysis and 16S rRNA gene clone libraries (Sievert et al., 1999; Giovannelli et al., 2013; Price R. et al., 2013; Sollich et al., 2017). There are to date only two studies on Paleochori Bay that involve deep-sequencing technology of the 16S rRNA marker gene, both focusing on a single vent (Bühring and Sievert, 2017; Sievert et al., 2022). A detailed structural analysis of the communities throughout Paleochori Bay and their links to patterns of fluid discharge is therefore still missing. Similarly, little is known about prokaryotic abundances, as the quantification of microbial communities both in Paleochori Bay and in the neighboring Spathi Bay has only been done on a limited number of samples (Sievert et al., 1999; Giovannelli et al., 2013; Callac et al., 2017; Fru et al., 2018).

In this study, we report the first in-depth characterization of the prokaryotic communities of Paleochori Bay using deep sequencing of the 16S rRNA marker gene as well as quantitative polymerase chain reaction (qPCR) quantification of the 16S rRNA gene and several functional genes. The study uses samples collected during 2 field expeditions in 2014 and 2019, summing to a total of 84 samples over 20 sediment cores, 2 microbial mats, and seawater. Our results provide a high-resolution description of the microbial communities inhabiting a wide range of habitats along hydrothermal gradients in the bay. Furthermore, we also discuss *in situ* interactions between the biosphere and the geochemical environment in the bay and review previous publications on the topic.

## Materials and methods

In this study, data collected during two different field campaigns are presented, called thereafter Saganaki and CarDHynAl datasets. The two datasets were produced using protocols with slight differences which are highlighted below. Both datasets describe microbial community composition using deep sequencing of the 16S rRNA marker gene, while qPCR analysis was performed only on the CarDHynAl dataset. A graphical summary of the Material and methods can be found in Supplementary material 1.

### Sampling

#### Saganaki dataset

Saganaki is a single vent site located ∼300 m offshore in Paleochori Bay (36.671490053°N, 24.516882251°E) at a water depth of 12 m (Figure 1B). It is characterized by temperatures up to 76.2°C in the shallow subsurface (~10 cmbsf), weak to moderate gas venting, and the colonization of the sediment surface by a white ∼1 cm thick microbial mat. Areas of flourishing seagrass surround the area of active venting, separated from the white mats by a transition zone (~1 m wide) of sediment where the burrowing activity of the mud shrimp *Calianassa truncata* is visible. In May 2014, scuba divers measured *in situ* temperatures and collected sediment cores and water samples. Sediments were cored with polycarbonate tubes and sealed underwater with rubber caps. Four sediment cores were collected along a 2 m transect, starting from the center of a white mat, through a transition zone, and ending in a seagrass-covered region (Figure 1D). A background area, devoid of seagrass and visually unaffected by venting, was also sampled. Sediment cores were immediately subsampled onshore by collecting 2 cm-thick slices in sterile falcon tubes. Subsamples were then stored and shipped on dry ice and then kept frozen at −80°C until processing. A seawater sample was also taken at less than 1 m depth in the vicinity of Saganaki. All cores and samples are listed in Supplementary material 2.

#### CarDHynAl dataset

In September 2019, a field campaign was organized for the CarDHynAl project to map and characterize temperature outflows (Puzenat et al., 2021) and microbial communities in Paleochori Bay. The sediments were sampled throughout Palaeochori Bay, from 3 to 10 m depth, and from a variety of visually different seafloor types such as background sand, bioturbation sand, ochre sand, white precipitates, zones covered with microbial mats, yellow sand, and black sand (Figure 1D). A hydrothermal hexagonal seafloor pattern in the south-east of the bay (36.672334236°N, 24.519504665°E; Figures 1B,C) was used to sample a high-resolution sediment transect across a zone of diffuse hydrothermal outflow. Sediment cores were taken using plexiglass tubes of 3.6 cm in diameter. Upon recovery of the cores, the sediment was immediately pushed out of the tubes and subsampled by using tip-sectioned syringes. Subsamples were stored at −20°C within the hour of sampling until further processing. Furthermore, 2 mat samples were collected using syringes (Figure 1B). All samples are listed in Supplementary material 2. Eleven fluid samples were also sampled around 5 cm below seafloor using a syringe connected to a short tubing (see list in Supplementary material 3). Temperatures were measured using a thermal blanket and multiple thermal probes as described in Puzenat et al. (2021).

### Fluid analyses

Within an hour after sampling, the fluids from the CarDHynAl dataset were split into two aliquots of 10 ml. Few grains of zinc acetate were added to the aliquot for anion analysis, and 0.3 ml of concentrated nitric acid was added to the aliquot for cation analysis. Samples were then kept at room temperature until analysis. Major anions were measured using Ion Chromatography (IC, Metrohm CompactIC), and major cations were measured using Inductively coupled plasma optical emission spectrometry (ICP-OES, Thermo Scientific iCAP 7,600).

### DNA extraction and sequencing

#### Saganaki dataset

Around 0.5 g of sand/sediment samples were homogenized using a sterile mortar and pestle, and bulk environmental DNA was extracted from both sediments and fluids according to the method of Mills et al. (2012). Negative controls were routinely used to confirm the absence of contamination during the process. Extracted DNA samples were then sent to Molecular Research DNA (Shallowater, TX, United States) for sequencing of the 16S rRNA gene using an Illumina Miseq platform with universal 515f and 806r primers (Caporaso et al., 2011). The list of all primers and PCR programs used in this study can be found in Supplementary material 4.

#### CarDHynAl dataset

DNA was extracted from ∼0.5 g of sediment/mat using the Dneasy^®^ PowerLyzer^®^ Power Soil Kit (Qiagen) following the manufacturer’s instructions and a FastPrep 24 Tissue Homogenizer (MP Biomedical). Negative controls were routinely run to assess contamination throughout the protocol. In order to produce amplicon libraries for sequencing, a 2-step amplification approach was used as described in Berry et al. (2011). First, triplicate PCRs were run on each sample using the HotStarTaq kit (Qiagen) and the 519F and 805R primers (see Supplementary material 4). After pooling of the triplicates and cleaning of the amplicons using AMPure XP beads (Beckman Coulter, Inc.), a second PCR was run to attach tags and sequencing adaptors to the PCR products. The final products were cleaned again using AMPure XP beads, and pooled equimolarly prior to sequencing on an Ion Torrent 7467 PGM machine at the University of Bergen.

### Sequence processing

After retrieval of the sequences, they were processed using an adaptation of the «alternative VSEARCH pipeline» (Rognes, 2021). In short, the two datasets were first separately trimmed of their primers using Cutadapt 3.2 (Martin, 2011), truncated at 220 bp and quality filtered at a max expected error of 1 for the Saganaki dataset and 2 for the CarDHynAl dataset using VSEARCH v.2.19.0 (Rognes et al., 2016). The reason for the difference is that Illumina produces higher quality sequences, allowing for a stricter cleaning process. Both datasets where then concatenated and processed in VSEARCH as follow: Dereplication and removal of singletons, clustering of OTUs at 97% similarity, *denovo* and reference-based chimera removal (using SILVA138.1 (Quast et al., 2013)), and finally mapping of the pre-dereplication sequences to the OTUs. The OTUs were further curated using LULU v.0.1.0 (Frøslev et al., 2017), prior to be given taxonomic assignments using the CREST4 LCA classifier (Lanzén et al., 2012) and the SILVA138.1 database. Finally, the remaining OTUs were run through a thorough decontamination process: Removal of OTUs with no domain assignments, contamination removal using the frequency approach in the *decontam* package (Davis et al., 2018), contamination removal using a list of known contaminants (Eisenhofer et al., 2019), and removal of low-abundance OTUs (Bokulich et al., 2013). The script used to process the sequences is available from https://github.com/MeinzBeur/LeMoineBauer-2022-Milos.

### qPCR

Quantitative PCR was used on the DNA extracted from the samples of the CarDHynAl dataset in order to quantify a selection of genes involved in various metabolic pathways. Several genes were successfully quantified: *aprA*, *dsrA* and *soxB* (sulfur cycle), *arrA* and *aoxB* (arsenic cycle), *nirK* and *nirS* (nitrogen cycle), and *mcrA* (CO2/methane cycle). For these genes, the standard curve allows to quantify down to ~10^3^ copies per gram of sediments. As well, two primer sets targeting separately the archaeal and bacterial 16S rRNA genes were used to quantify prokaryotic abundances. However, primer sets targeting the archaeal *amoA*, bacterial *amoA*, and *hzo* (nitrogen cycle) did not show any product during preliminary PCR screening on a selection of samples. They were therefore not used for qPCR and the genes were considered to be not present in the dataset. Furthermore, primer sets targeting *arsC, arxA* and *arsB* (arsenic cycle) and *psbA* (algal photosynthesis) did not produce any amplicon during preliminary PCR testing on a selection of samples either. However, due to the lack of a positive control, we cannot rule out the possibility of unsuccessful PCR protocol optimization for these genes. Finally, qPCR assays of the *nifH* gene (nitrogen fixation) and *pmoA* (methane/CO2 cycle) showed abnormal amplification pattern (likely due to low amplification efficiency) and were therefore not further used. The genes were however present in our samples. All qPCRs were run on a StepOne™ Real-Time PCR System (ThermoFisher Scientific) using the Quantitect SYBR green PCR kit (Qiagen). Gene copy numbers are given as copies per gram sediment but are not scaled for the number of copies per cell, and therefore do not represent true cell abundance. Total 16S rRNA copy numbers (or prokaryotic 16S rRNA numbers) were calculated as the sum of bacterial and archaeal 16S rRNA gene copies. A summary of all successfully and failed primer sets used, along with primer sequences, thermocycler programs and comments can be found in Supplementary material 4.

### Statistical analysis

The different cores were positioned and assigned to seafloor types by carefully comparing *in situ* observations and published photomosaics (Martelat et al., 2019; Puzenat et al., 2019a,b). All statistical analyses were made in R v.4.1.2 (R Core Team, 2021), with the recurrent use of the phyloseq package (McMurdie and Holmes, 2013) and the ggplot2 package (Wickham et al., 2022). The alpha diversity was assessed using the Shannon diversity index, as it is robust to differences in sequencing depth. The index was computed at the OTU level. Barplots of the compositions are shown at the family, phylum, and domain level. For the rest of the study, the analyses were made at the family taxonomic level, as this showed to be a good balance between keeping relevant taxonomic information and decreasing the noise created by having more and rarer taxa in the dataset. We followed the principle of Compositional Data Analysis (Aitchison, 1986; Pawlowsky-Glahn et al., 2015; Gloor et al., 2017). After clustering at the family level, 63% of our OTU table were zeros, and we therefore decided to add 1 as a pseudo count to all counts of the table as this zero-imputation method has been shown to be more efficient than other methods for very sparse data (i.e., with high proportion of zeros; Lubbe et al., 2021). The OTU table was then clr-transformed, centered, and subjected to principal component analysis based on singular value decomposition. The scores of the form biplot were then extracted and plotted in R using a modified version of geom_link2 (Pedersen, 2021). In order to describe the different seafloor types, we computed balances using the selbal R-functions (Rivera-Pinto et al., 2018) as such approach has been suggested to be more suitable for compositional data than simple differential abundance analysis (Quinn et al., 2021). In our study, the algorithm identifies two groups of microbial taxa of which the ratio will statistically differ between samples belonging to different seafloors (For more explanation, see Supplementary Data 8). The script used to process the sequences is available from https://github.com/MeinzBeur/LeMoineBauer-2022-Milos.

## Results and discussion

### Seafloor types of Paleochori Bay

The seafloor of Paleochori Bay exhibits a wide range of distinct visual diversity (Figure 1D). These differences can easily be linked to the influence of the hydrothermal activity, and each seafloor type can be constrained by its temperature range (Puzenat et al., 2021). As temperature is known to be a major structuring variable of microbial communities in hydrothermal systems (Lagostina et al., 2021), we decided to classify our samples according to the seafloor type they were taken from. This resulted in 8 different seafloor types, described here and shown in Figure 1D. (i) Background sediments are gray/beige and appear unimpacted by hydrothermal activity, with temperatures in cores at Saganaki and in the CarDHynAl dataset similar to that in local seasonal seawater (19°C and 23–25°C, respectively). (ii) Seagrass patches are covered by the sparse growth of *Cymodocea nodossa*. Despite being only 2 meters away from a venting place, the seagrass core at Saganaki shows temperatures close to the background seafloor, only rising from 19 to 21°C in the upper 20 cm of the sediments. Pore water chemistry from Saganaki’s seagrass and background cores show strong similarities, except for slightly higher dissolved iron, As(III), dissolved organic carbon and total dissolved nitrogen in seagrass patches (data not shown). (iii) The bioturbation seafloor has the same color as the background seafloor but is easily identified by the burrowing activity of the mud shrimp *Calianassa truncata*. The zone is typically present as a band of up to a couple of meters width that surrounds the white hydrothermal patches but can also be found in bigger patches disconnected from visible hydrothermal activity (Puzenat et al., 2021). At Saganaki, the increase in temperature from 23.1 to 34.2°C in the upper 20 cm is in agreement with the measurements from Puzenat et al. (between 21.7 and 41.4°C at 35 cm depth). The temperatures at the seafloor interface are close to seawater temperatures. (iv) Ochre patches are characterized by the brown color of the sand, due to the precipitation of iron and manganese oxides (Wenzhöfer et al., 2000), and can be found surrounding warmer seafloor areas (e.g., white and mat patches, described below), or in broad and isolated patches. Temperatures at 35 cm depth vary between 42.0 and 54.7°C (Puzenat et al., 2021). (v) White patches can be recognized by the presence of a superficial white dust precipitate, and were mainly observed in the eastern zone of Paleochori Bay, near the hexagon. This seafloor type is under stronger hydrothermal influence than the previous types described, and the fluids sampled in this seafloor show notably a higher silicon content than the Background ones (Supplementary Data 3). Nevertheless, we did not observe intense venting zones or thick microbial mats on the seafloor. It is however likely that a gradual transition can be observed from white patches to mat patches (see below). Puzenat et al. measured temperatures at 35 cm depth between 52.8 and 73°C with an average of 66.8°C (Puzenat et al., 2021). (vi) Mat patches exhibit dark gray/black sand and are characterized by white fluffy microbial mats of up to a couple of centimeters that accumulate in the ripple marks. It appears in the vicinity of strongly degassing black patches or very hot yellow patches. It is therefore subject to intense hydrothermal venting, as shown by the fluid chemistry (Supplementary material 3). Temperatures for this environment are very heterogeneous (Puzenat et al., 2021), but measurements for our two cores show temperatures of 54.3 and 57.6°C at 10 cm and rising to 59.8 and 71.3°C by 20 cm, respectively. (vii) Black patches are found in association with intense degassing sites, where no bacterial mat grows anymore. The temperatures are between 30 and 40°C near the surface and reach 90°C at 10 cm depth. The chemical analysis of the fluid however shows very little difference compared to seawater, except for the increase in silicon concentrations (Supplementary Data 3). This suggests limited hydrothermal fluid flow at this degassing location, and/or that there is a strong recharge of seawater at that location, as suggested previously (Wenzhöfer et al., 2000; Yücel et al., 2013). (viii) Yellow patches are recognizable by their yellow/orange sand and are also subject to very high hydrothermal impact, exhibiting similar temperature as the black patches (around 90°C at 10 cm depth). However, the degassing is much weaker and the pore fluid chemistry showed very strong hydrothermal influence (Supplementary Data 3). Notably, we measured an arsenic concentration of 84.3 μM.

### Microbial community structure of the different seafloor types

A compositional data principal component analysis [CoDa-PCA; (Aitchison, 1983; Pawlowsky-Glahn et al., 2015)] of all 81 seafloor samples provided a visual assessment of microbial community structures from OTUs to phylum level. The general clustering pattern remained consistent at each taxonomic level (Supplementary material 5), but here we present only the family level analyses, apart from the Shannon diversity index which was computed using OTUs. The family level was chosen for the following reasons: (1) We wanted to decrease the noise that would be present in OTU level analyses, (2) it separates the abundant families *Arcobacteraceae*, *Sulfurimonadaceae* and *Sulfurovaceae* which have a distinct distribution pattern and would be clustered together at a higher taxonomic level, (3) it allows to reduce the dissimilarity observed between the CarDHynAl and Saganaki datasets that could arise from differences in sample preparation and primer sets used, and (4) it decreases the occurrences of zero counts in the dataset that need to be accounted for in the compositional analysis.

**Figure 2:**
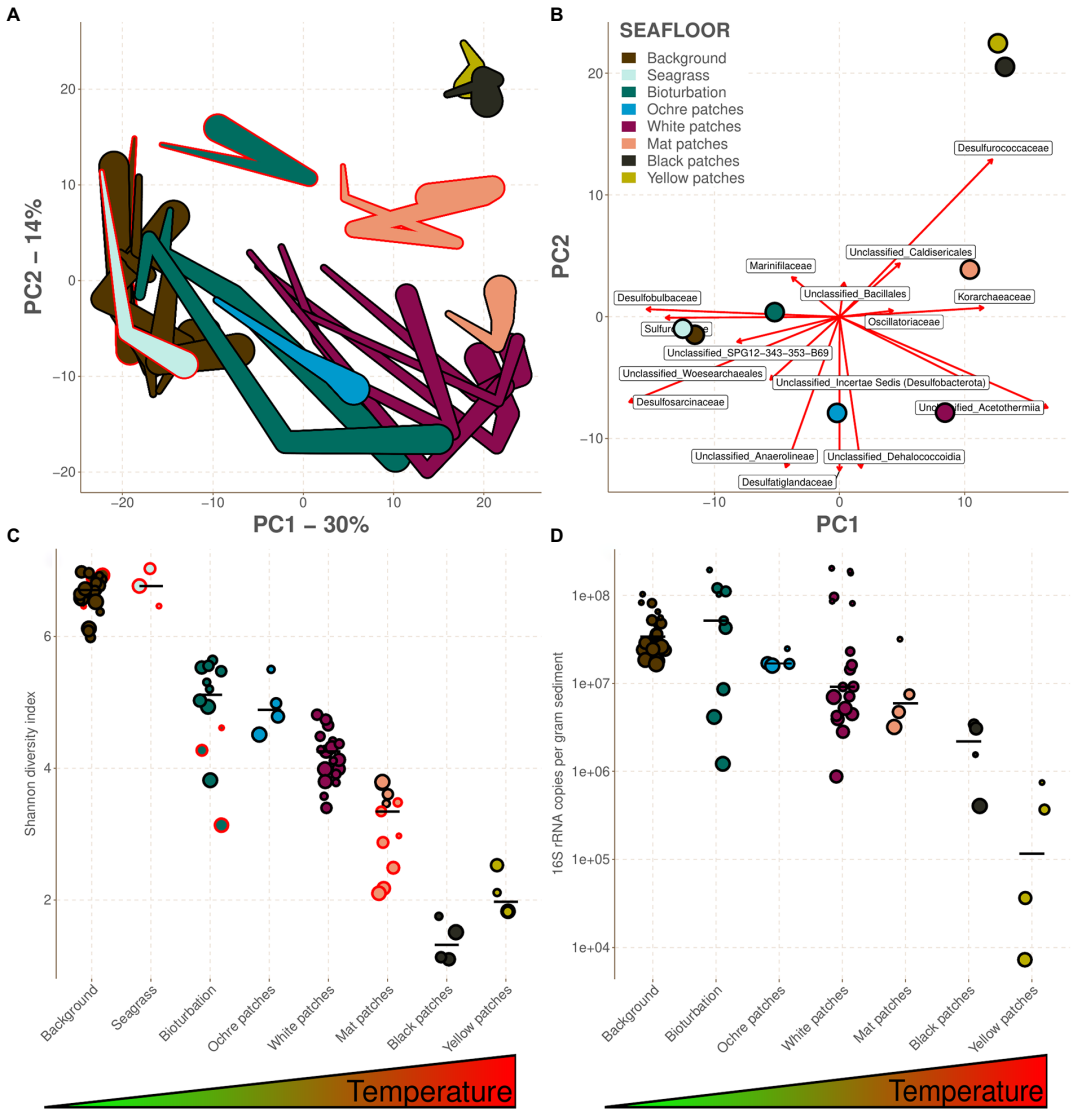
Heterogeneity between the different seafloor types. The color legend as given in B is true for the whole figure. Samples from the Saganaki dataset have a red border, while the ones from the CarDHynAl dataset have a black border. **(A)** Form CoDa-biplot, representing 44% of the variance. It approximately reflects the Aitchison distances between the 16S rRNA composition of the sample at the family taxonomic level. Each linked samples represent a core, with the width of the link being proportional to depth of the sample. **(B)** Covariance CoDa-biplot where the center of each seafloor type is shown, as well as the taxa involved in the balances described in the text. **(C)** Shannon diversity index of each sample grouped per seafloor type. **(D)** Absolute prokaryotic abundance as copies of 16S rRNA per gram of sediment quantified by qPCR. For **(C,D)**, the seafloor types are ordered by increasing temperature/hydrothermal influence, and the bars represent the median.

On the resulting ordination, PC1 explains 30% of the variance, PC2 explains 14% and PC3 explains 9%. PC1 is segregating the samples along the first part of the temperature gradient, with the cold background and seagrass samples on the left side, and then in order bioturbation, ochre patches, and white patches when moving along PC1 (Figure 2A). On the warmer end of PC1, PC2 then separates the remaining cores, with the mat patches first and finally the yellow and black patches in the upper right corner. The correlation between PC1 and temperature is also visible within each core, with the shallower and colder end of each core being almost systematically aiming to the left side of PC1. This pattern is lost in the background and seagrass seafloors where there is almost no temperature gradient throughout the cores. The CoDa-PCA analysis also shows that the samples from the Saganaki dataset tend to separate from the samples of the CarDHynAl dataset along PC3 (Supplementary material 5), as well as PC2 for the Bioturbation and Mat patches (Figure 2A). While the use of different primer sets (see Supplementary material 6 for the *in silico* primer analysis) are likely influencing the separation, real biological heterogeneity within each seafloor type is also possible. Despite these differences, the Saganaki dataset shows the same correlation between PC1 and temperature, strengthening our observation of temperature and hydrothermal activity being the main structuring factor (in agreement with Dando et al., 1995b; Sievert et al., 1999; Sollich et al., 2017). This hydrothermal impact on microbial communities is also observable on the diversity, as shown by the decrease of the Shannon diversity index along the temperature gradient (Figure 2C; Sievert et al., 1999). Furthermore, absolute 16S rRNA concentrations decrease from around 10^8^ to 10^4^ copies per gram sediments when getting closer to the vents (Figure 2D). Our estimates are however around an order of magnitude lower than in previous studies that used direct cell counts (Sievert et al., 1999 and Giovannelli et al., 2013), likely due to the different methods used (Lloyd et al., 2013). Finally, the archaeal fraction of the community composition increases with temperature (Supplementary Data 7; Sievert et al., 2000a), which is also observed in other hydrothermal systems (Lagostina et al., 2021). The hydrothermal impact is also highlighted by fluid/seawater mixing models at Milos, which suggest that microbial metabolic strategies often shift with mixing ratio, and therefore temperature (Lu et al., 2020).

The following sections describe the prokaryotic communities inhabiting each type of seafloor. However, the ordination and the diversity analysis highlighted a strong similarity between the communities inhabiting the seagrass and background seafloors, the bioturbation and ochre patch seafloors, and the black and yellow patch seafloors (Figures 2A,C). Therefore, these seafloor types will be described together from now on. This also allows us to use the selbal R-functions on all groups, while the low number of samples for some seafloor types would have otherwise prevented it.

#### Background and seagrass patches

**Figure 3:**
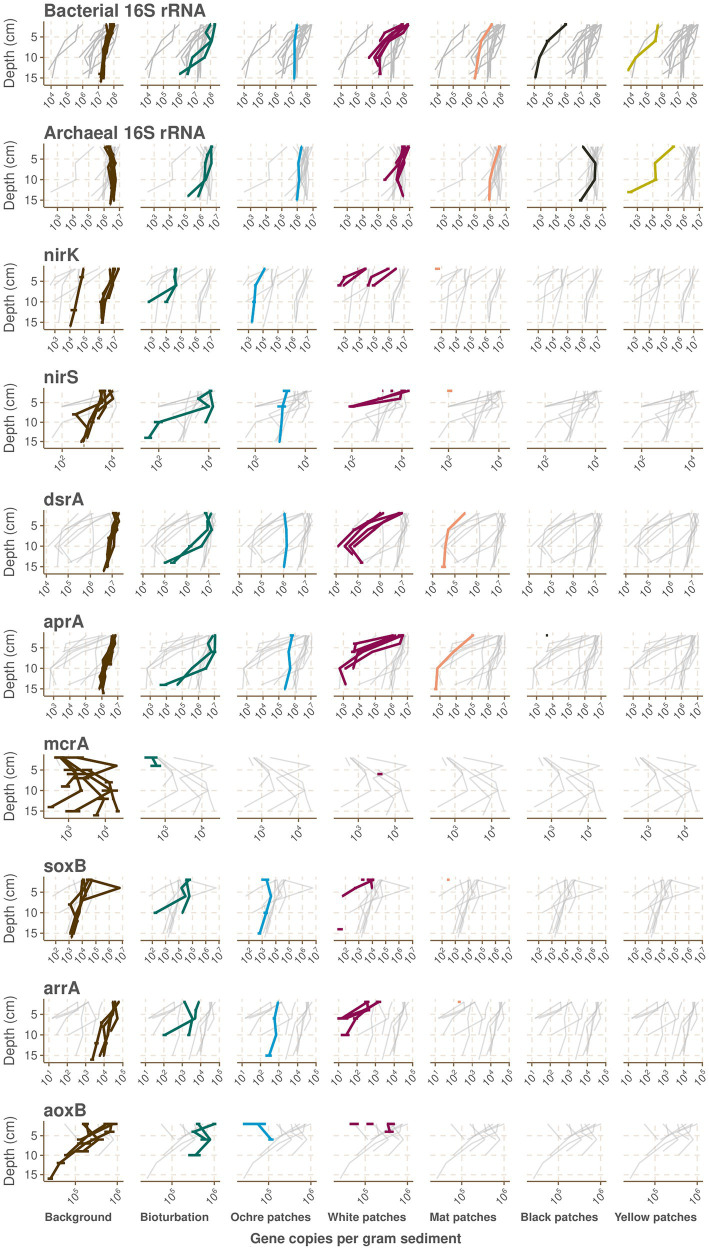
Absolute abundances per gram of sediments of various genes in the samples from the CarDHynAl dataset measured by qPCR. Samples are plotted according to their depth and their seafloor type. For each plot, the gray background lines represent the values for this gene in the other seafloor types. Horizontal error lines represent the standard deviation of qPCR triplicates.

The background seafloor possesses the second highest absolute prokaryote abundance of our dataset after the bioturbation samples (Figure 2D), with 16S rRNA copy numbers ranging from 1.66e+7 to 1.03e+8 copy.g^−1^ sediment. Deeper samples tend to have lower absolute abundances than the shallow ones, which is mainly linked to the decrease in Bacteria (Figure 3). Based on these qPCR measurements, Archaea copies account for 1.0 to 22.1% of the total prokaryote gene copies and are less influenced by depth than Bacteria (Figure 3). No quantitative data are available for the seagrass seafloor type but considering the similarity with the background seafloor type one might expect similarly high 16S rRNA copy numbers.

**Figure 4:**
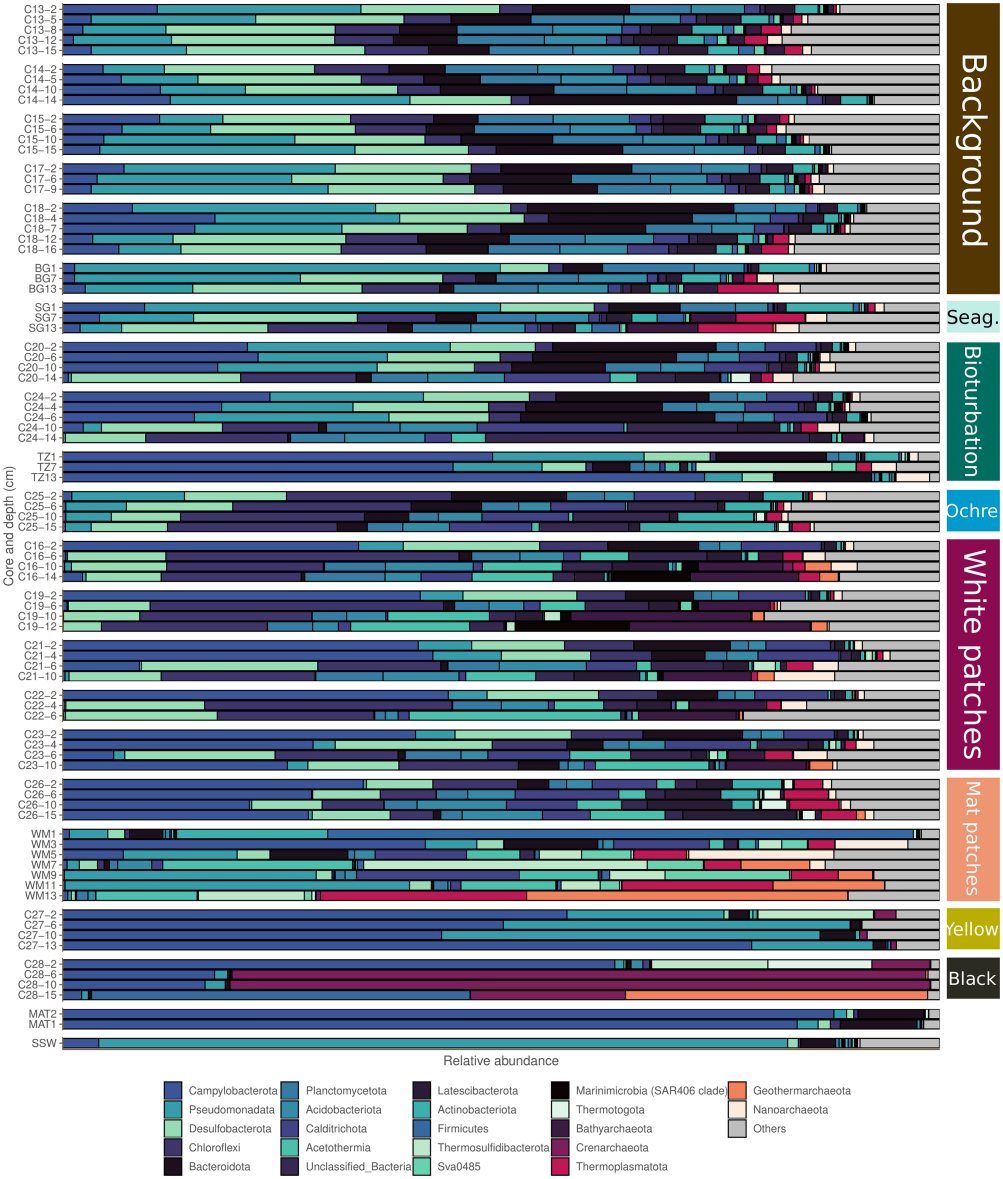
Microbial community compositions of the different seafloor types based on 16S rRNA analysis. The barplots show phyla that represent at least 10% of the community in at least one sample from the dataset. The color code to the right for the seafloor types is as in Figure 2.

**Figure 5:**
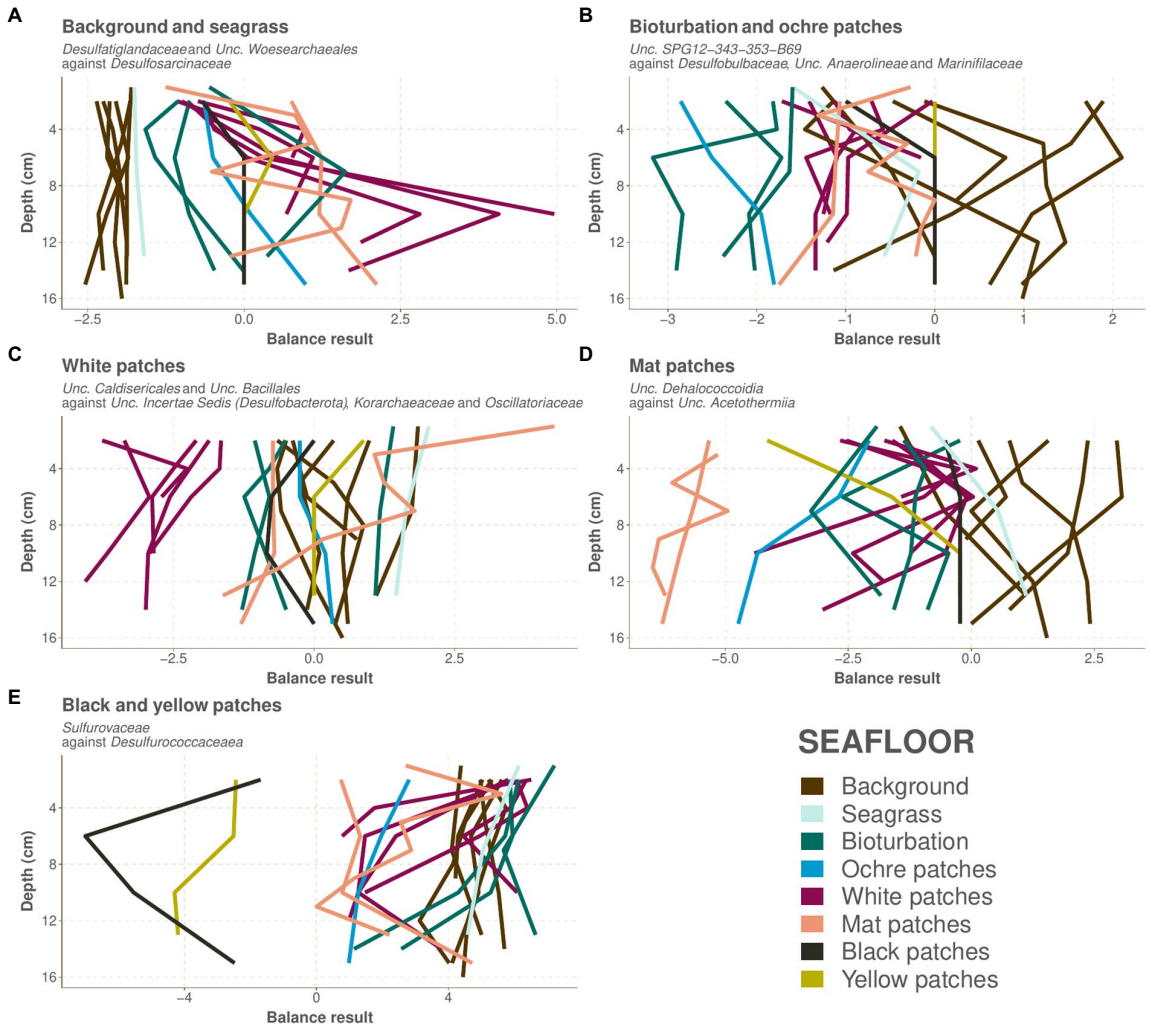
Values for the balances selected by the selbal algorithm for the separation of each group of seafloors. The values are plotted against depth, and each path represents one core, colored as in Figure 2. The color code for the seafloor types is as in Figure 1. **(A)** Background and seagrass, **(B)** bioturbation and ochre patches, **(C)** white patches, **(D)** mat patches, **(E)** black and yellow patches.

The background and seagrass cores are the most diverse of our samples (Figure 2C). Their prokaryotic community composition at the phylum taxonomic level is shown in Figure 4 and at the family level in Supplementary Material 7. The composition of the seagrass core is very similar to the background core from the Saganaki dataset (Figure 4). Background and seagrass samples can be segregated from the others using the balance of *Desulfatiglandaceae* and *Unclassified_Woesearchaeales* against *Desulfosarcinaceae*, with balance values between −2.54 and − 1.60 (median − 1.97) for the background and seagrass samples, and between −1.58 and 4.96 (median 0.00) for the others (Figure 5A; Supplementary Material 8).

#### Bioturbation and ochre patches

The bioturbation zone has the highest prokaryote abundance of our dataset (Figure 2D), with 16S rRNA copy numbers ranging from 1.22e+6 to 1.95e+8 copy.g^−1^ sediment. The deeper samples exhibit the lower counts, with a decrease of around 2 orders of magnitude in the top 15 cm (Figure 3). Based on these qPCR measurements, Archaea copies represent between 1.8 and 20.3% of the total prokaryote gene copies. The core from the ochre patch shows little variation in prokaryote copy numbers, with a total of 1.6e+7 to 2.48e+7 16S rRNA copy.g^−1^ sediment. Neither Bacteria nor Archaea copy numbers decrease in the top 15 cm, and therefore the relative abundance of Archaea remains between 6.0 and 7.7% (Figure 3). The low Archaea content in the bioturbation and ochre patches is similar to the background and seagrass seafloor type, suggesting a rather low hydrothermal impact on these zones.

The CoDa-PCA analysis shows that the bioturbation and ochre patches act as a transition zone, with the deepest samples clustering with the warmer seafloors, while the shallower samples are similar to the background and seagrass samples (Figure 2A). This is also observed in the spatial distribution of these patches, which are often surrounding warmer white and mat patches. The bioturbation and ochre patches cores are less diverse than the background and seagrass samples (Figure 2C). Their prokaryotic community composition at the phylum taxonomic level is shown in Figure 4 and at the family level in Supplementary material 7. The high heterogeneity of the community within and between each bioturbation and ochre patch core (Figure 2A), as well as its similarity with the center of the dataset (centers plotted close to the origin of PC1 and PC2 on Figure 2B) makes it more difficult to characterizes this seafloor types using selbal. The resulting balance opposes *Unclassified_SPG12-343-353-B69* to *Desulfobulbaceae, Unclassified_Anaerolineae* and *Marinifilaceae*, with balance values between −3.17 and −1.60 (median −2.06) for the bioturbation and ochre patch samples, while all other samples have values between −1.75 and 2.10 (median −0.42; Figure 5B; Supplementary material 8). Major compositional differences can be seen between the bioturbation cores from the Saganaki and the CarDHynAl datasets. We suggest that the Saganaki core exhibits warmer temperatures, as supported by the presence of taxa mainly found in cores from warmer seafloor types (Figure 4 and Supplementary material 7), its lower diversity compared to the CarDHynal core, and the geographical location of the Saganaki bioturbation zone that surrounds a mat patch, which is warmer than the white patch the CarDHynAl bioturbation zone surrounds. The composition of the ochre patch is similar to a subcomposition of the bioturbation patch, where *Sulfurovaceae* has been removed. The presence of iron oxidizing *Zetaproteobacteria* in ochre patches at the nearby Spathi Bay has been shown through qPCR (Callac et al., 2017), but our sequencing data do not support this in our ochre core, with only few *Zetaproteobacteria* sequences detected in the surface of the core (data not shown).

#### White patches

In the cores from the white patches, the 16S rRNA gene copy numbers range from 8.74e+5 to 2.03e+8 copy.g^−1^ sediment, with a strong decrease with depth (Figure 2D). The decrease is particularly visible for Bacteria, and therefore the relative abundance of Archaea increases with depth, with values between 2.6 and 55.9% of the total prokaryote copies according to qPCR data (Figure 3).

The white patches show a further decrease in diversity compared to previously described seafloor types. Their prokaryotic community composition at the phylum taxonomic level is shown in Figure 4 and at the family level in Supplementary material 7. The balance *Unclassified_Caldisericales* and *Unclassified_Bacillales* against *Unclassified_Incertae Sedis (Desulfobacterota)*, *Korarchaeaceae* and *Oscillatoriaceae* segregates the white patch samples from the rest, with values from −4.07 to −1.66 (median −2.85) for the white patches and from −1.62 to 4.27 (median 0.00) for the others (Figure 5C; Supplementary material 8). Puzenat et al. (2021) reports some very strong gradients of temperature within the white patches, which is also reflected in the change of composition along the cores. For example, the logratio of *Sulfurovaceae* over *Unclassified_Bathyarchaeota* systematically decreases with depth in each core, turning negative around 4–6 cm depth.

#### Mat patches

The cores from the mat patch show lower absolute 16S rRNA abundance than cores from colder seafloor types, with values going from 3.2e+6 to 4.1e+7 copy.g^−1^ sediments (Figure 2D). Both Bacteria and Archaea copy numbers decrease with depth, and the relative abundance of Archaea goes from 6.4 to 25.6% of the total prokaryote copies according to qPCR data.

Mat patches samples have a lower diversity than the colder seafloors. Their prokaryotic community composition at the phylum taxonomic level is shown in Figure 4 and at the family level in Supplementary material 7. The CoDa-PCA analysis (Figure 2A) and the barplots reveal that the 2 cores exhibit different compositions. For the selbal analysis, we removed the sample WM1 (1 cm deep in the Saganaki core), due to the complete lack of similarity with the rest of the core (We suggest that the surface community of the sediments may be influenced by the surroundings; see discussion in the “Sulfur cycling” section). The selbal analysis selected the balance *Unclassified_Dehalococcoidia* against *Unclassified_Acetothermiia* to segregate this seafloor type from the others, with balance values between −6.48 and −4.95 (median −6.00) for the mat patch samples and between −4.74 and 3.06 (median −0.36) for the others (Figure 5D; Supplementary material 8). We observe some differences between the Saganaki core and the CarDHynAl core, with for example a much stronger decrease in the logratio of *Arcobacteraceae* over *Unclassified_Geothermarchaeota* in the Saganaki core, along with a lower diversity (Figure 2C). This could be linked to different core temperatures, as well as the different types of fluids at the sampling location: The Saganaki core shows the presence of low salinity fluids in the deep section while the CarDHynAl core had high salinity fluids (Supplementary Data 3). Based on clone libraries, differences in bacterial and archaeal community composition between high and low salinity cores has already been shown (Price R. et al., 2013). It is difficult to compare the results we obtain with the ones from Price et al., but taken together these observations suggest some heterogeneity in the mat patches that cannot be constrained by only a few cores.

#### Black and yellow patches

These seafloor types have the lowest absolute 16S rRNA abundance of the dataset, with values ranging from 4.0e+5 to 3.4e+6 copy.g^−1^ sediment for the black patch and from 7.2e+3 to 7.5e+5 copy.g^−1^ sediment for the yellow patch (Figure 2D). While both Archaea and Bacteria copies decrease with depth in the yellow patch, Archaea remains stable in the top 15 cm of the black patch (Figure 3). According to qPCR data, Archaea 16S rRNA copies account for 34.1 to 99.1% of the total in black patches, and 3.7 to 42.9% in yellow patches.

Black and yellow patches are the seafloor types with the lowest diversity within our dataset. Their prokaryotic community composition at the phylum taxonomic level is shown in Figure 4 and at the family level in Supplementary material 7. The CoDa-PCA analysis (Figure 2A) clusters both seafloor types together, but the barplots reveal that the 2 cores exhibit different compositions. Notably, the logratio of *Sulfurimonadaceae* over *Desulfurococcaceae* is positive in the yellow patch, while it is negative in the black patch. The selbal algorithm selected the balance *Sulfurovaceae* against *Desulfurococcaceae* to discriminate these two seafloor types from the others, with balance values between −7.01 and −1.69 (median −3.35) for the samples from the black and yellow patches and between 0.00 and 7.21 (median 4.58) for the others (Figure 5E; Supplementary material 8). The fact that *Desulfurococcaceae* are almost only detected in these seafloor types plays an important role in the balance. *Stetteria hydrogenophila*, a member of this family, has been previously isolated at Milos in sediments of 107°C (Jochimsen et al., 1997).

#### Microbial mats

Two different types of microbial mats were sampled in this study, with MAT1 growing on a rock in a flow of hydrothermal fluids, while MAT2 was a 2 cm thick mat lying on top of the Mat patches (as already described in Dando et al., 1995b). The microbial mats are entirely devoid of Archaea (only 0.1% of the community in the mat sample attached to the rock). Both samples present the same community pattern, with a composition dominated by *Campylobacterota* (around 80% of the community), as well as some *Bacteroidetes* (Figure 4). This composition is similar to mats observed in deep sea hydrothermal systems, such as at Loki’s Castle Vent Field (Stokke et al., 2015). Both samples contain the families *Sulfurimonadaceae* and *Arcobacteraceae*, but *Sulfurovaceae* is only present in the mat sample from the rock. The genus *Arcobacter* has already been found in the mats previously (Sievert et al., 1999), and has been suggested to possibly play a key role in their formation (Sievert et al., 2007). However, the composition described here is in contradiction to earlier descriptions who showed the dominance of *Achromatium volutans* in the mats, a genus not detected in our study (Dando et al., 1995b; Fitzsimons et al., 1997). Similarly, Fitzsimons et al. (1997) reported the presence of *Thiobacillus* sp. in the mat, which we did not detect. Diatoms have also been reported in the mats, but we did not investigate these here (Sievert et al., 1999).

#### Seawater

The seawater sample of the Saganaki dataset is completely different from any other sample taken in this study (Figure 4). The taxa with highest relative abundance belong to Clade I of SAR11 and the AEGEAN-169 marine group (both *Alphaproteobacteria*), the SAR86 clade and the *Halieaceae* (both *Gammaproteobacteria*) and the *Puniceicoccaceae* (*Verrucomicrobiota*).

### Microbial metabolic capabilities in Paleochori sediments

In addition to our extensive sequencing data of the prokaryotic communities, we have also quantified several functional genes using qPCR on the CarDHynAl dataset. In the following section, we discuss these results in light of previous studies on bioenergetic landscapes (Lu et al., 2020), most probable numbers of various functional groups (Sievert et al., 1999), and reaction rates (Dando et al., 1995b; Bayraktarov et al., 2013; Gilhooly et al., 2014; Houghton et al., 2019) to investigate some metabolic capabilities of the communities. It is however important to note that the detection of functional genes in the DNA extraction of a sample merely suggests the potential for the community to perform the metabolic pathway but does not imply its use. We divide the discussion into the following geochemical processes relevant to this ecosystem: Organic carbon remineralization, nitrogen cycling, sulfur cycling, iron cycling, methanogenesis, and arsenic cycling.

#### Organic carbon remineralization

In sediments, microbes typically use organic matter (OM) deposited from the seawater as an electron donor. The process oxidizes the organic matter through the reduction of various electron acceptors, which will be used in sequence according to the amount of energy released: Oxygen first, then nitrate, manganese, iron, sulfate, and carbon dioxide. The more organic matter present in the sediments, the faster these electron acceptors will be depleted, and the penetration of oxygen in sediments is therefore closely linked to organic matter content (Middelburg, 2019). In hydrothermal settings, the pattern is however more complex as rising fluids are also a source of electron donors, allowing the growth of autotrophic species that will in turn be a new source of OM for remineralization. At Milos, oxygen has been shown to penetrate the sediments with only a few centimeters at the venting sites and a few millimeters to no penetration at all when leaving the vents (Sievert et al., 1999; Wenzhöfer et al., 2000; Yücel et al., 2013). The deeper penetration at vents is however suggested to be due to short-scaled seawater recharge patterns rather than lower OM content. Indeed, Dando et al. (1995b) showed that there is more total organic carbon (TOC) at the vents compared to background sediments, which they suggest reflects an increase in microbial biomass at the venting sites. However, Giovannelli et al. (2013) rather show a decrease in microbial biomass and abundance when leaving the vents, and therefore hypothesize that the higher TOC at vents is due to a decrease in remineralization. This effect is likely enhanced by the inhibition of sulfate reduction under the acidic conditions created by the hydrothermal fluids (Bayraktarov et al., 2013). Our qPCR results of bacterial and archaeal 16S rRNA genes confirm the previous observations that microbial abundance decreases with increasing temperature (Figures 2D, 3). Organic matter remineralization is nevertheless likely a important process in Paleochori Bay, as suggested by the regular detection of putative heterotrophs such as *Acidimicrobiia*, *Anaerolineae*, *Bacteroidia*, *Fusobacteriia*, *Thermodesulfobacteria* and *Thermotogae* (this study; Sievert et al., 2000a,b, 2022; Giovannelli et al., 2013; Price R. et al., 2013; Price R.E. et al., 2013).

#### Nitrogen cycling

The *nirK* gene, involved in heterotrophic and autotrophic denitrification, has highest absolute abundance (around 10e+7 copies per gram sediment) in the shallowest samples of the background cores, and shows decreasing trends with depth and increasing temperature (Figure 3). There is however some variance, with for example the background core C18 that exhibits concentrations 2 orders of magnitude lower than the other background cores, suggesting some heterogeneity even within the background seafloor. As well, the white patches have higher concentrations of *nirK* than the colder bioturbation and ochre patch seafloor types close to the surface, however *nirK* falls below detection limit at sediment depth below 6 cm in the white patches. *NirK* is then only detected at 2 cm depth in the mat patches and is absent in the very hot black and yellow patches. However, the *nirK* gene has been shown to divide into two phylogenetically distinct clades, and our primers amplify only a clade composed of *Alphaproteobacteria* plus few *Gamma*- and *Betaproteobacteria* (Helen et al., 2016). Most *nirK* gene diversity is found in the other clade, which include members of *Bacteroidetes*, *Chloroflexi*, *Nitrospirae*, *Firmicutes*, *Actinobacteria*, *Planctomycetes*, and several archaeal lineages. This can potentially explain why we do not detect the *nirK* gene in warmer seafloor. Our quantification of the *nirS* gene shows a similar pattern, but once more our primer set has been shown to omit numerous taxa, including members of the *Campylobacterota* which are dominant in the hydrothermally influenced sediments and known to reduce nitrate and nitrite (Murdock and Juniper, 2017). Nitrate/nitrite reduction is likely still happening at higher temperatures, as supported by models showing that the energy released by sulfide oxidation coupled to nitrite reduction could support much of the chemolithotrophic primary production at the venting site (Lu et al., 2020). Such chemolithotrophic nitrate-reducing sulfur-oxidizing bacteria have been isolated from Milos in the past, but they are mesophilic and therefore unlikely to grow directly at the venting site (Kuever et al., 2002). The identification of potential denitrifier in our dataset using the 16S rRNA sequences is difficult, as the process is potentially performed by a wide phylogenetic range of microbes (Helen et al., 2016). Nevertheless, most *Campylobacterota* can use nitrate as a terminal elecron acceptor, and we also detect the presence in the bioturbation and ochre patch seafloors of the *Calditrichaceae* family which are moderately thermophilic and some members are known to grow by respiring nitrate (Bonch-Osmolovskaya and Kublanov, 2021).

We do not detect in our 16S rRNA dataset any *Nitrosopumilaceae*, a family known for the potential of its members to oxidize ammonia aerobically (Qin et al., 2017). Consistently, no bacterial or archaeal *amoA* gene was detected in the CarDHynAl dataset. However, we detect high relative abundances of *Geothermarchaeaceae* in the mat patch core from the Saganaki dataset. While very little is known about this family, they also belong to the *Thaumarchaeota*/*Nitrososphaeraeota* phylum. This, along with the observation that ammonia oxidation is exergonic under these conditions (Lu et al., 2020), could suggest that *Geothermarchaeaceae* might be involved in this process.

#### Sulfur cycling

Sulfur cycling in marine sedimentary environments is a complex network of connected biotic and abiotic reactions driven largely by the marine sulfate reservoir as the ultimate sulfur source. These processes transform sulfur species between the oxidized (sulfate) and reduced (sulfide) end members, along with numerous intermediate valence compounds (reviewed in Jørgensen et al., 2019). *DsrA* and *aprA*, involved in sulfate reduction, display a similar distribution pattern as *nirK*, with around 10e+7 copies per gram sediment in the shallowest background cores, and a decrease with increasing depth and temperature (Figure 3). Most of the previous studies at Milos on sulfate reduction have shown a similar trend. Notably, the same *dsrA* qPCR trend was found in the neighboring Spathi Bay, albeit with much higher gene copy concentration compared to this study (Callac et al., 2017). Sulfate reduction rates were found to peak in the upper 2 cm, although with no relation to the distance from the vent (Dando et al., 1995b), while other isotopic studies found an increase of sulfate reduction when leaving the vents (Bayraktarov et al., 2013; Houghton et al., 2019). Only one study reported that no obvious isotopic signature for biotic sulfur cycling could be found (Gilhooly et al., 2014), but they suggest that this could be linked to a lack of available TOC for remineralization in their samples, highlighting once more the spatial heterogeneity within each type of seafloor at Paleochori Bay. Most probable number studies found an increase of sulfate reducing bacteria away from the vent (Sievert et al., 1999). However, we find members of the *Desulfobacterota* phylum, of which many members can reduce sulfate using *dsrAB* (Waite et al., 2020), throughout our dataset. While lower pH has been shown to inhibit sulfate reduction at Milos, some sulfate reducers can also adapt to life at low pH (Bayraktarov et al., 2013). In support, the thermophilic sulfate-reducing bacterium *Desulfacinum hydrothermale* was isolated from pH 5 sediments (Sievert and Kuever, 2000). However, in our study, the family *Syntrophobacteraceae*, to which *D. hydrothermale* belongs, is mainly detected in seafloors with colder temperatures and pH close to neutral. In our core from the black patch, we find the genus *Staphylothermus*, a sulfur reducing hyperthermophilic Archaeon belonging to the family *Desulfuroccocaceae* also isolated from Milos (Supplementary Data 7; Arab et al., 2000; Hao and Ma, 2003).

In areas of hydrothermal activity, the advective influx of reduced sulfur species can also fuel the growth of sulfur oxidizing organisms (Sievert et al., 2008, 2022). At Milos, the fluids contain abundant H_2_S (Fitzsimons et al., 1997), and the sulfidic zone has been shown to reach or nearly reach the seafloor, except directly at the vent where seawater microcirculation allows for oxygen to penetrate a few centimeters (Wenzhöfer et al., 2000; Yücel et al., 2013). As a result, many studies at Milos have reported the presence of bacteria able to oxidize sulfide (SOB). The most striking feature is the presence of cotton-like white microbial mats whether lying on the mat patches or attached to rocks where fluids come out (Dando et al., 1995b). We find that these mats consist to around 80% of *Arcobacteraceae*, *Sulfurimonadaceae*, and *Sulfurovaceae,* which contain the well-known marine SOB genera *Arcobacter*, *Sulfurimonas*, and *Sulfurovum*, respectively (Wirsen et al., 2002; Inagaki et al., 2003, 2004; Sievert et al., 2007). In the sediments, the energy densities of sulfide oxidation are very variable, suggesting highly localized processes, but with higher potential for autotrophy in the more hydrothermally influenced areas where sulfide oxidation coupled to the reduction of oxygen, nitrate, and/or nitrite could support much of the chemolithotrophic primary production (Lu et al., 2020; Sievert et al., 2022). As well, sulfur isotopic analyses suggest that H_2_S is oxidized by microbes close to the venting zone (Houghton et al., 2019). However, our qPCR analysis of the *soxB* gene shows that the gene is most abundant in the background samples, decreasing with depth and increasing temperature to finally be only present in the shallowest sample of the mat patch seafloor type (Figure 3). The presence of SOB in the background and bioturbation seafloor types was already shown using most probable number dilution series (Brinkhoff et al., 1999; Sievert et al., 1999), and isotopic studies then suggested that they could recycle the sulfide produced by sulfate reducer (Houghton et al., 2019). The rapidly changing geochemical conditions at Milos, for example due to waves and storms, could shift the direction of the reaction, explaining why we observe both sulfate reducers and sulfide oxidizers in the same samples (Figure 4; Supplementary material 7). The absence of *soxB* genes in our warmer cores is likely the result of primer specificity. Indeed, we measure very low gene abundance of *soxB* in our samples (around 10e+4 in the background and the seagrass seafloors) in comparison to the high abundance of bacterial 16S rRNA copies (around 10e+7 in the same seafloors), and *in silico* analysis shows that our *soxB* primers do not target *Campylobacterota* (Supplementary Data 6). Our sequencing data show a change in the family distribution within the *Campylobacterota* phylum: While *Sulfurovaceae* dominate in the background, seagrass and white patches, they are then replaced by *Arcobacteraceae* and *Sulfurimonadaceae* in the mat, yellow and black patches (Supplementary material 7). The bioturbation seafloor type shows all three families, and our ochre patch core seems mostly devoid of *Campylobacterota*, despite the *soxB* gene being detected there. Recently, the *Sulfurovaceae* family was similarly not detected in an *in-situ* carbon fixation experiment in the mat patches (Sievert et al., 2022). However, community compositions of 0–1 cmbsf hydrothermal and background sediment where *Sulfurovaceae* is a major part of the composition in both types of sediments have been described (Giovannelli et al., 2013). They report that *Epsilonproteobacteria* (now *Campylobacterota*) represent 60% of each prokaryotic community, which is much more than what we measured. However, superficial sediment communities are likely impacted by microbial mats and seawater communities, and are therefore highly dissimilar to deeper samples as seen in some of our cores (data not shown). The genus *Thiomiscrospira,* and the strain *Thiomicrospira* sp. Milos-T1 isolated at Milos, has been suggested to be a major actor of sulfur oxidation in Paleochori Bay (Brinkhoff et al., 1999), but these results were not supported in a recent study (Sievert et al., 2022), and in our dataset the family *Thiomicrospiraceae* is mainly present only in the yellow patch composition. Similarly, the family of the sulfur oxidizing bacteria *Halothiobacillus kellyi* isolated at Milos (Sievert et al., 2000b) represents at best 0.9% of any sample.

Finally, the family *Desulfocapsaceae* and the genus *Desulfocapsa* known for sulfur disproportionation (Finster et al., 1998) are detected in the background cores (Supplementary material 7). This reaction had been shown to be exergonic in most niches around hydrothermal systems (Alain et al., 2022), but we cannot confirm its use at Milos with our dataset.

#### Iron cycling

We did not directly investigate the presence of genes involved in iron reduction in our study. Nevertheless, dissimilatory iron reducing bacteria have been found at Milos in the colder seafloor types, seemingly correlated to Fe(III) concentrations (Sievert et al., 1999). It is however difficult to identify which organisms are responsible for iron reduction. The iron reducer *Deferrisoma palaeochoriense* has been isolated from Paleochori Bay (Pérez-Rodríguez et al., 2016), but we find *Deferrisomataceae*, its family, to represent a maximum of 0.3% of the community of any sample in our dataset. Similarly, the *Deferribacteraceae* (Huber and Stetter, 2015), the *sva1033* family of the *Desulfobacterota* (supposed to perform dissimilatory iron reduction (Wunder et al., 2021)), and the *Archaeoglobaceae* (to which *Geoglobus* belongs (Kashefi et al., 2002; Slobodkina et al., 2009)) represent at best 0.2% of the community in any sample. Among the taxa with higher relative abundance, the order *Actinomarinales,* representing up to 14.6% of the community in the ochre patches, have been shown to have a gene cluster related to the acquisition of Fe(III) (López-Pérez et al., 2020).

#### Methanogenesis

*McrA* in our study is detected only in the background and in 3 single samples in the bioturbation and white patch seafloors. In the background cores, the gene abundance peaks between 5 and 10 cm. This would suggest that methanogenesis would only occur at a specific horizon. While Callac et al. also found the highest *mcrA* copies in their background reference sediments, they also detect the gene in the brown and white sediments and do not have an abundance peak at a few centimeters depth (Callac et al., 2017). Based on the data available, the relevance of methanogenesis at Milos remains elusive.

#### Arsenic cycle

Arsenic is a compound that is ubiquitous in the marine biosphere, where it is poisonous to most organisms due to its chemical similarity with phosphate (Bidlack, 2002). However, some prokaryotes have developed strategies to detoxify from arsenic, and sometimes even use it as an energy source (Páez-Espino et al., 2009; Tsai et al., 2009). In Paleochori Bay, the hydrothermal venting discharges high concentrations of arsenic, with concentrations of up to 78 μM previously measured in low salinity fluids (Price R.E. et al., 2013). At the yellow patch location of our C27 core, we measured concentrations of arsenic of 84 μM in high salinity fluids (Supplementary material 3), and observed under scanning electron microscopy the precipitation of arsenic oxides and orpiment (As_2_S_3_) coating the sand grains as described previously (Supplementary material 3; Price R.E. et al., 2013; Godelitsas et al., 2015). We investigated the adaptation of prokaryotes to arsenic by quantifying the *arrA* (Arsenate respiratory reductase) and *aoxB* (Arsenite oxidase) genes involved in energy production. We also tried to amplify *arxA,* also involved in energy production, and *arsB* and *C,* involved in detoxification, but with no avail. Both *arrA* and *aoxB* showed similar abundance pattern, with highest concentrations found in the shallow background sediments, and a decrease with increasing depth and temperature (Figure 3). The *arrA* gene is not detected beyond the shallowest sample in the mat patch seafloor, while the *aoxB* gene is not present beyond the white patches. We do not detect these genes in the core from the yellow patch, where high concentrations of arsenic are measured. While seemingly counter-intuitive, similar results were found in the nearby Spathi Bay, where higher concentrations of *aoxB*, *arrA*, *arsB*, *acr3-1* and *acr3-2* were found away from the venting zone (Fru et al., 2018). The authors suggest that in the vicinity of the vents, the acidic fluids and high sulfur concentrations effectively trap arsenic into orpiment by precipitation. The lack of such arsenic sink further away correlates with the increase of arsenic-related genes. Also, arsenic-resistance genes are much more abundant than arsenic-metabolism genes, suggesting the ability of the community to rapidly adapt to arsenic surges in the system (Fru et al., 2018). Similar to our qPCR analysis, our 16S rRNA sequencing data does not show any clear response of the community composition to the high arsenic concentrations in the yellow patch. This observation matches the results from Callac et al., who did not find arsenic to be a major selector for carbon fixation genes in the nearby Spathi Bay (Callac et al., 2017). Price et al. found some *aioA* (other name of *aox*) sequences in the mat patches, but the clones, belonging to *Alphaproteobacteria* and *Betaproteobacteria,* showed little overlap with their 16S rRNA data (Price R. et al., 2013), suggesting that these are not major parts of the community composition. This is consistent with the models showing that reactions using As(V) as an electron acceptor can provide only very little energy at Milos (Lu et al., 2020). However, we found that As(III) is present in low concentrations in the shallows to middle depths in the bioturbation core from Saganaki (data not shown). In this core we also find the presence of the *Sulfurospirillaceae* family, to which the Fe(III) and As(V) respiring bacterium *Sulfospirillium barnesii* belongs (Stolz et al., 1999), and the presence of arsenic could therefore be linked to the desorption of arsenic upon the dissolution of solid iron phases (Herbel and Fendorf, 2006).

## Conclusion and future perspectives

**Figure 6:**
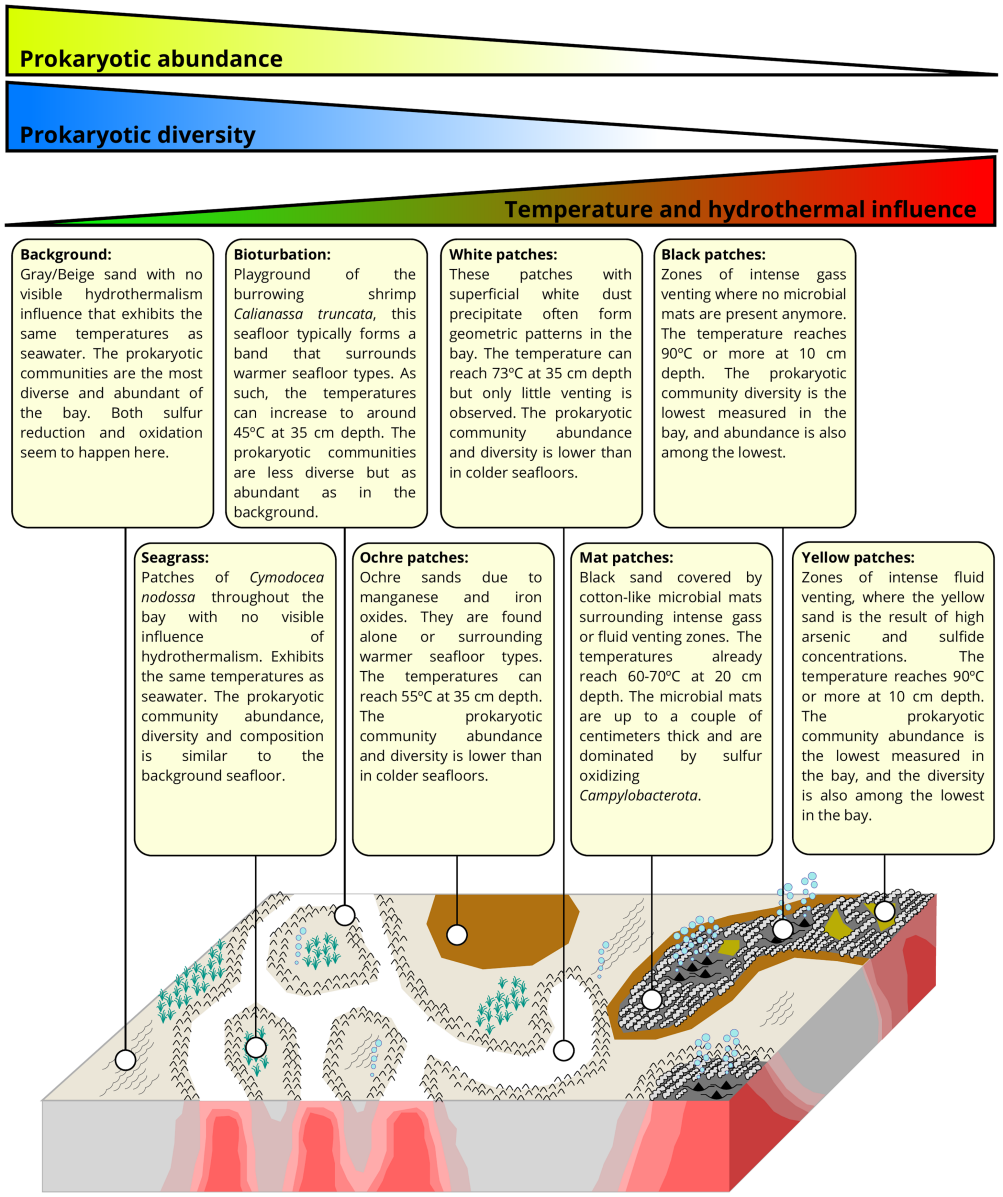
Description of the various seafloors observed in Paleochori Bay, along with the general trends of abundance, diversity, and composition of the prokaryotic communities (modified from the hydrothermal distribution model for Milos in Puzenat et al. (2021)).

The present study has provided a significant upgrade in our understanding of the prokaryotic communities in the Paleochori Bay of Milos. For the first time we present a thorough description of the visual diversity of seafloor types present in the bay and the prokaryotic communities inhabiting them (Figure 6). Our results show the strong impact of hydrothermal activity on community composition, abundance, diversity, and metabolic potential. It should however be noted that we observe some heterogeneity within seafloor types, and hence our samples are unlikely to cover the entire span of microbial diversity inhabiting the bay.

Furthermore, several aspects of the ecosystem are still poorly investigated, and we find the following potential research subjects very relevant to further increase our understanding of the microbial communities inhabiting Paleochori Bay. (i) The physical, chemical, and microbiological heterogeneity of some seafloor types are not well constrained. Notably we present only one core taken from the ochre patches while they represent a massive surface area. As well, the difference between low-salinity and high-salinity communities in the mat patches needs further investigation. (ii) A major difference between deep and shallow hydrothermal systems is the possibility for phototrophic organisms to play a role in the food chain. During our sequence decontamination protocol, we removed in some samples up to 8% of reads assigned to chloroplasts. This mainly happened in the colder seafloor types, suggesting higher importance of phototrophy there. However, the importance of phototrophy in the ecosystem is yet to be investigated. (iii) While we have presented here a substantial dataset of 16S rRNA gene sequences, the general understanding of the microbial communities would highly benefit from transcriptomic studies. This could for example help understanding why sulfur reducers and oxidizers are present in the same samples, and also constrain the temporal impact of tides on the communities. (iv) Sulfur oxidizing microbial mats are common in hydrothermal systems. However, due to its temporal instability, the mats at Paleochori Bay are ideal to investigate their development and growth, allowing for *in situ* experiments on mat growth that would be otherwise difficult in the deep sea. (v) Viruses are known to be key player in marine ecosystems (Suttle, 2007; Rohwer et al., 2009; Sime-Ngando, 2014), and are likely to play a strong ecological and evolutionary role in deep hydrothermal systems (Ortmann and Suttle, 2005; Castelán-Sánchez et al., 2019). In shallow systems, Manini et al. (2008) described a rather low viral abundance, but there is to our knowledge no other investigation of the viral community in shallow hydrothermal systems. Milos, with its high temporal variability, would be an ideal place to study and monitor the virus-host dynamics at play. (vi) Milos is a major natural discharge of arsenic and therefore an ideal place to investigate how organisms cope with this highly poisonous compound. Godelitsas et al. (2015) and our study identified potentially biogenic arsenic-sulfide filaments and spirals (Supplementary Data 3), which have previously been suggested to result from the mineralization of hyphae (Dekov et al., 2013). Further investigations on arsenotrophy and arsenic detoxification based on cultures or molecular approaches could potentially help developing new arsenic bioremediation approaches.

## Data availability statement

The data presented in the study are deposited in the European Nucleotide Archive database, project accession number PRJEB56441.

## Author contributions

SL, VP, AS, TB, J-EM, JEs, JA, PP, and OV participated in the sampling campaign at Milos. SL, G-SL, and SG did the laboratory work. SL, VP-G, and JEg analyzed the data. SL, G-SL VP-G, JEg, JA, and SJ actively participated in the writing of the article. All coauthors read and approved the manuscript.

## Funding

Funding for the Saganaki dataset and JPA and G-SL was provided by NSF grants OIA-0939564 to the Center for Dark Energy Biosphere Investigations (C-DEBI). The CarDHynAl project was partially funded by INSU-CNRS Tellus and Syster Projects to JEs (2016) and J-EM (2018), and with partial support by RAMONES, funded by the European Union’s Horizon 2020 Research and Innovation Program, under grant agreement number 101017808 (to JEs and PN). The laboratory work was supported by the Trond Mohn Foundation and the University of Bergen through the Centre for Deep Sea research (grant TMS2020TMT13). VP-G and JEg are supported by the Spanish Ministry of Science and Innovation and the European Regional Development Fund [grant numbers PID2021-125380OB-I00 and PID2021-123833OB-I00 (MCIN/AEI/FEDER)].

## Conflict of interest

The authors declare that the research was conducted in the absence of any commercial or financial relationships that could be construed as a potential conflict of interest.

## Publisher’s note

All claims expressed in this article are solely those of the authors and do not necessarily represent those of their affiliated organizations, or those of the publisher, the editors and the reviewers. Any product that may be evaluated in this article, or claim that may be made by its manufacturer, is not guaranteed or endorsed by the publisher.

## Supplementary material

The Supplementary material for this article can be found online at: https://www.frontiersin.org/articles/10.3389/fmicb.2022.1060168/full#supplementary-material
