# Supplementary material for: Structure and metabolic potential of the prokaryotic communities from the hydrothermal system of Paleochori Bay, Milos, Greece
Source: Front Microbiol. 2023 Jan 6;13:1060168. doi: 10.3389/fmicb.2022.1060168 (PMC9852839; doi:10.3389/fmicb.2022.1060168)
Supplement: Supplementary file 1 [file Data_Sheet_1.pdf]

Supplementary data to: Structure and metabolic potential of the prokaryotic communities from the hydrothermal system of Paleochori Bay, Milos, Greece.

Sven Le Moine Bauer, Guang-Sin Lu, Steven Goulaouic, Valentine Puzenat, Anders Schouw, Thibaut Barreyre, Vera Pawlowsky-Glahn, Juan José Egozcue, Jean-Emmanuel Martelat, Javier Escartin, Jan P. Amend, Paraskevi Nomikou, Othonas Vlasopoulos, Paraskevi Polymenakou, Steffen Leth Jørgensen

Supplementary material 1: Overview of the study

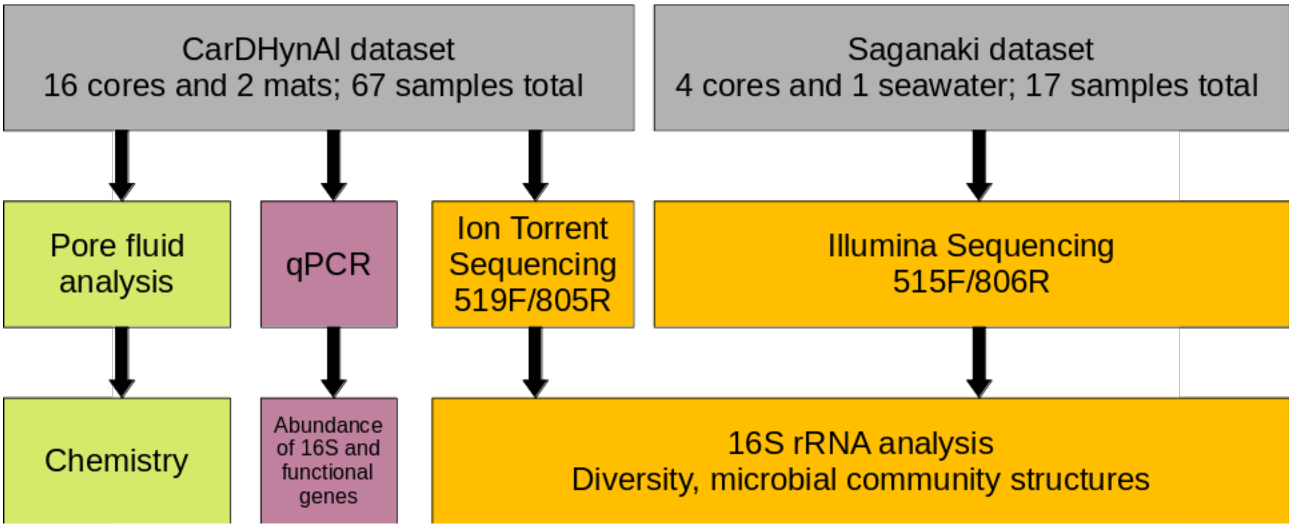

Supplementary material 2: Summary of the cores and samples taken

Note that samples C25_0, C26_0, TZF and WMF were not used in the analysis.

Sample	Dataset	Core	Depth (cm)	Seafloor type	Latitude	Longitude
C13_2	Cardhynal	C13	2	Background	24.519504665	36.672334236
C13_5	Cardhynal	C13	5	Background	24.519504665	36.672334236
C13_8	Cardhynal	C13	8	Background	24.519504665	36.672334236
C13_12	Cardhynal	C13	12	Background	24.519504665	36.672334236
C13_15	Cardhynal	C13	15	Background	24.519504665	36.672334236
C14_2	Cardhynal	C14	2	Background	24.519514183	36.672365952
C14_5	Cardhynal	C14	5	Background	24.519514183	36.672365952
C14_10	Cardhynal	C14	10	Background	24.519514183	36.672365952
C14_14	Cardhynal	C14	14	Background	24.519514183	36.672365952
C15_2	Cardhynal	C15	2	Background	24.519522796	36.672392643
C15_6	Cardhynal	C15	6	Background	24.519522796	36.672392643
C15_10	Cardhynal	C15	10	Background	24.519522796	36.672392643
C15_15	Cardhynal	C15	15	Background	24.519522796	36.672392643
C16_2	Cardhynal	C16	2	White patches	24.519532002	36.672423018
C16_6	Cardhynal	C16	6	White patches	24.519532002	36.672423018
C16_10	Cardhynal	C16	10	White patches	24.519532002	36.672423018
C16_14	Cardhynal	C16	14	White patches	24.519532002	36.672423018
C17_2	Cardhynal	C17	2	Background	24.519471291	36.672326531
C17_6	Cardhynal	C17	6	Background	24.519471291	36.672326531
C17_9	Cardhynal	C17	9	Background	24.519471291	36.672326531
C18_2	Cardhynal	C18	2	Background	24.519437830	36.672317823
C18_4	Cardhynal	C18	4	Background	24.519437830	36.672317823
C18_7	Cardhynal	C18	7	Background	24.519437830	36.672317823
C18_12	Cardhynal	C18	12	Background	24.519437830	36.672317823
C18_16	Cardhynal	C18	16	Background	24.519437830	36.672317823
C19_2	Cardhynal	C19	2	White patches	24.519396893	36.672306002
C19_6	Cardhynal	C19	6	White patches	24.519396893	36.672306002
C19_10	Cardhynal	C19	10	White patches	24.519396893	36.672306002
C19_12	Cardhynal	C19	12	White patches	24.519396893	36.672306002
C20_2	Cardhynal	C20	2	Bioturbation	24.519460912	36.672387038
C20_6	Cardhynal	C20	6	Bioturbation	24.519460912	36.672387038

C20_10	Cardhynal	C20	10	Bioturbation	24.519460912	36.672387038
C20_14	Cardhynal	C20	14	Bioturbation	24.519460912	36.672387038
C21_2	Cardhynal	C21	2	White patches	24.519445255	36.672396272
C21_4	Cardhynal	C21	4	White patches	24.519445255	36.672396272
C21_6	Cardhynal	C21	6	White patches	24.519445255	36.672396272
C21_10	Cardhynal	C21	10	White patches	24.519445255	36.672396272
C22_2	Cardhynal	C22	2	White patches	24.519431053	36.672404907
C22_4	Cardhynal	C22	4	White patches	24.519431053	36.672404907
C22_6	Cardhynal	C22	6	White patches	24.519431053	36.672404907
C23_2	Cardhynal	C23	2	White patches	24.519421835	36.672409993
C23_4	Cardhynal	C23	4	White patches	24.519421835	36.672409993
C23_6	Cardhynal	C23	6	White patches	24.519421835	36.672409993
C23_10	Cardhynal	C23	10	White patches	24.519421835	36.672409993
C24_2	Cardhynal	C24	2	Bioturbation	24.519413654	36.672415033
C24_4	Cardhynal	C24	4	Bioturbation	24.519413654	36.672415033
C24_6	Cardhynal	C24	6	Bioturbation	24.519413654	36.672415033
C24_10	Cardhynal	C24	10	Bioturbation	24.519413654	36.672415033
C24_14	Cardhynal	C24	14	Bioturbation	24.519413654	36.672415033
C25_0	Cardhynal	C25	0	Ochre patches	24.512942329	36.673089297
C25_2	Cardhynal	C25	2	Ochre patches	24.512942329	36.673089297
C25_6	Cardhynal	C25	6	Ochre patches	24.512942329	36.673089297
C25_10	Cardhynal	C25	10	Ochre patches	24.512942329	36.673089297
C25_15	Cardhynal	C25	15	Ochre patches	24.512942329	36.673089297
C26_0	Cardhynal	C26	0	Mat patches	24.512954022	36.673087666
C26_2	Cardhynal	C26	2	Mat patches	24.512954022	36.673087666
C26_6	Cardhynal	C26	6	Mat patches	24.512954022	36.673087666
C26_10	Cardhynal	C26	10	Mat patches	24.512954022	36.673087666
C26_15	Cardhynal	C26	15	Mat patches	24.512954022	36.673087666
C27_2	Cardhynal	C27	2	Yellow patches	24.519284508	36.673007234
C27_6	Cardhynal	C27	6	Yellow patches	24.519284508	36.673007234
C27_10	Cardhynal	C27	10	Yellow patches	24.519284508	36.673007234
C27_13	Cardhynal	C27	13	Yellow patches	24.519284508	36.673007234
C28_2	Cardhynal	C28	2	Black patches	24.516351243	36.673795029
C28_6	Cardhynal	C28	6	Black patches	24.516351243	36.673795029
C28_10	Cardhynal	C28	10	Black patches	24.516351243	36.673795029
C28_15	Cardhynal	C28	15	Black patches	24.516351243	36.673795029

MAT1	Cardhynal	MAT	NA	Mat	24.515712111	36.674058097
MAT2	Cardhynal	MAT	NA	Mat	24.516154429	36.674089557
BG1	Saganaki	BG	1	Background	~24.516882251	~36.671490053
BG7	Saganaki	BG	7	Background	~24.516882251	~36.671490053
BG13	Saganaki	BG	13	Background	~24.516882251	~36.671490053
SG1	Saganaki	SG	1	Seagrass	~24.516882251	~36.671490053
SG7	Saganaki	SG	7	Seagrass	~24.516882251	~36.671490053
SG13	Saganaki	SG	13	Seagrass	~24.516882251	~36.671490053
SSW	Saganaki	SSW	NA	Seawater	~24.516882251	~36.671490053
TZ1	Saganaki	TZ	1	Bioturbation	~24.516882251	36.671490053
TZ7	Saganaki	TZ	7	Bioturbation	~24.516882251	~36.671490053
TZ13	Saganaki	TZ	13	Bioturbation	~24.516882251	~36.671490053
TZF	Saganaki	TZ	NA	Bioturbation	~24.516882251	~36.671490053
WM1	Saganaki	WM	1	Mat patches	~24.516882251	~36.671490053
WM3	Saganaki	WM	3	Mat patches	~24.516882251	~36.671490053
WM5	Saganaki	WM	5	Mat patches	~24.516882251	~36.671490053
WM7	Saganaki	WM	7	Mat patches	~24.516882251	~36.671490053
WM9	Saganaki	WM	9	Mat patches	~24.516882251	~36.671490053
WM11	Saganaki	WM	11	Mat patches	~24.516882251	~36.671490053
WM13	Saganaki	WM	13	Mat patches	~24.516882251	~36.671490053
WMF	Saganaki	WM	NA	Mat patches	~24.516882251	~36.671490053

Supplementary material 3: Chemistry

This section presents the chemistry of the porewater sampled with the CarDHynAl dataset (**Table 1**). **Figure 1** then presents pictures and SEM observations of C27 containing high arsenic content.

Table 1: Chemistry of the fluids sampled with the CarDHynAl dataset. Al to Sr were measure with ICP-OES, while chloride, bromide and sulphate were measured on IC.

	Al	As	B	Ba	Ca	Co	Cu	Fe	K	Mg
	ppb	ppb	ppb	ppb	ppm	ppb	ppb	ppb	ppm	ppm
C13 (Background)	ND	ND	5133.80	28.14	455.35	ND	24.82	ND	429.34	1398.87
C14 (Background)	ND	ND	4989.14	31.81	451.69	ND	25.08	ND	429.05	1391.34
C15 (Background)	ND	ND	5052.00	35.27	455.34	ND	ND	ND	433.04	1399.01
C16 (White patch)	ND	126.09	6751.84	70.39	611.20	ND	26.35	ND	586.47	1316.71
C21 (White patch)	ND	ND	5690.23	33.24	515.46	ND	25.57	ND	494.22	1366.68
C22 (White patch)	ND	102.03	6613.44	57.69	599.31	ND	20.22	ND	576.07	1322.76
C23 (White patch)	ND	ND	8026.35	83.01	726.74	ND	21.86	ND	711.44	1261.66
C26 (Mat patch)	ND	ND	31813.02	1274.45	1534.76	ND	ND	507.58	2699.59	357.39
C27 (Yellow patch)	357.52	6319.17	60822.79	2735.17	2941.46	ND	20.89	5188.80	5416.00	142.09
C28 (Black patch)	ND	ND	6465.70	140.94	476.67	ND	27.99	ND	548.49	1282.08

	Mn	Na	Ni	P	Pb	Si	Sr	Chloride	Bromide	Sulphate
	ppb	ppm	ppb	ppb	ppb	ppb	ppb	ppm	ppm	ppm
C13 (Background)	167.30	11947.96	ND	ND	ND	596.11	8472.52	23609	76.1	3209
C14 (Background)	44.91	11930.92	ND	16.34	ND	480.97	8427.86	21586	75.6	3291
C15 (Background)	108.05	11995.65	ND	39.62	ND	816.08	8496.14	23626	76.6	3241
C16 (White patch)	988.80	12111.95	ND	0.26	ND	21060.79	9607.74	23164	78.4	3205
C21 (White patch)	403.55	12064.69	ND	ND	ND	8012.35	8859.28	23628	77.6	3293
C22 (White patch)	918.79	12100.41	ND	ND	ND	20017.86	9484.19	23057	78.3	3233
C23 (White patch)	1606.08	12289.22	ND	ND	ND	34588.04	10506.45	23455	78.0	3156
C26 (Mat patch)	4076.47	13828.65	ND	ND	ND	51394.80	24769.30	29391	77.8	989
C27 (Yellow patch)	10361.21	23190.41	ND	ND	61.66	86934.11	45310.65	48558	125.3	552
C28 (Black patch)	334.11	11515.37	ND	15.91	ND	16172.72	8836.03	22738	69.0	2712

Figure 1: Orpiment and arsenic oxide precipitations in C27 (Yellow patch) seen under SEM. In order, (1) Orpiment precipitation covers the right side of this quartz grain, (2) Zoom onto orpiment, with a salt cristal in the top right corner, (3) Orpiment precipitation on quartz, with some arsenic oxide cristals also present, and (4) Orpiment tubular structures showing similarities to biogenic iron stalks.

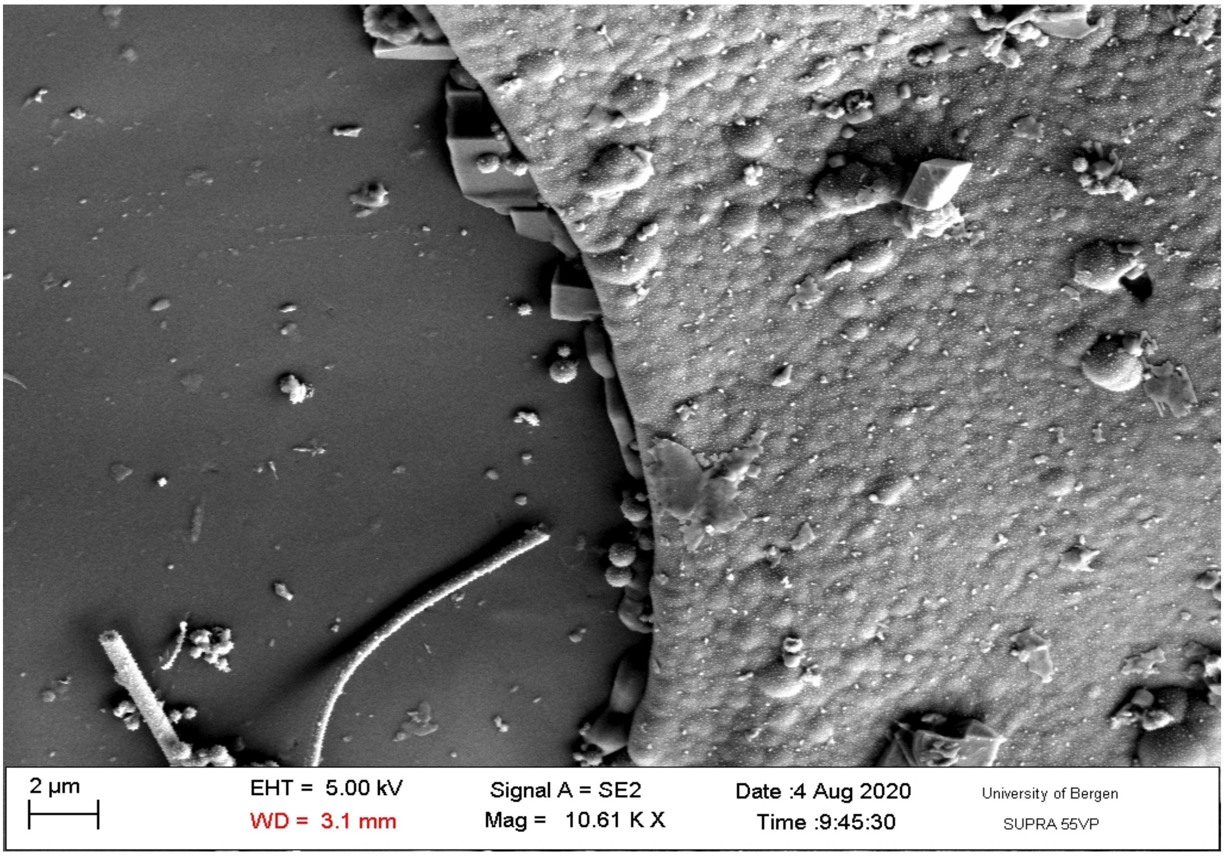

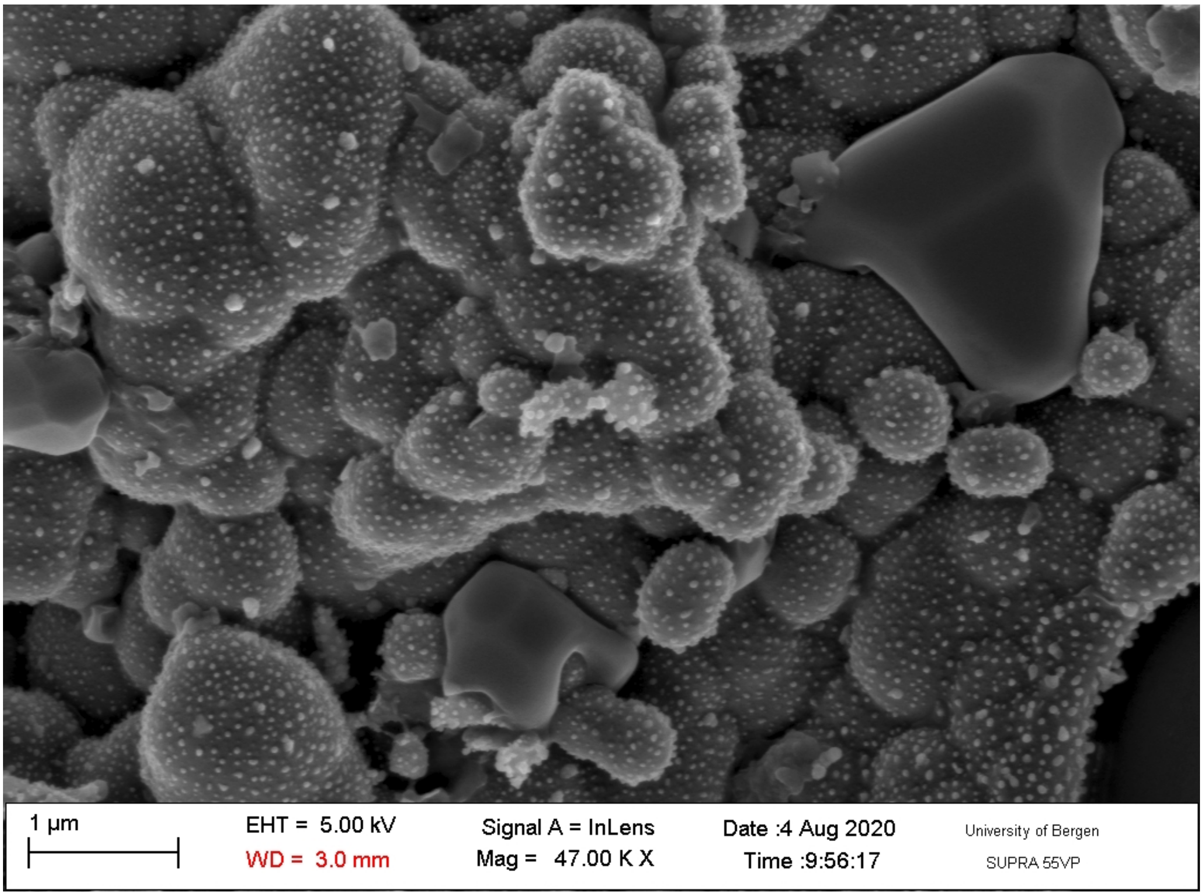

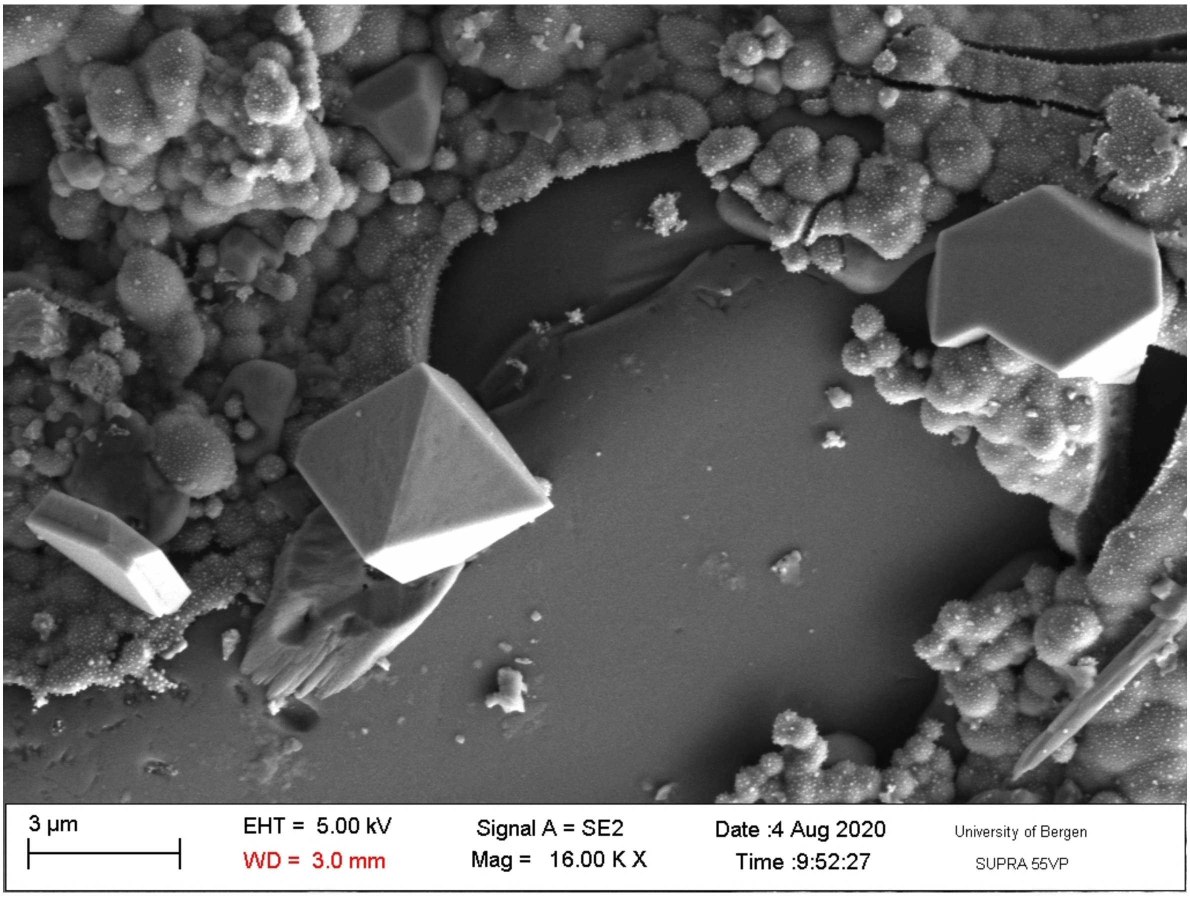
3 µm
EHT = 5.00 kV
WD = 3.0 mm
Signal A = SE2
Mag = 16.00 K X
Date :4 Aug 2020
Time :9:52:27
University of Bergen
SUPRA 55VP

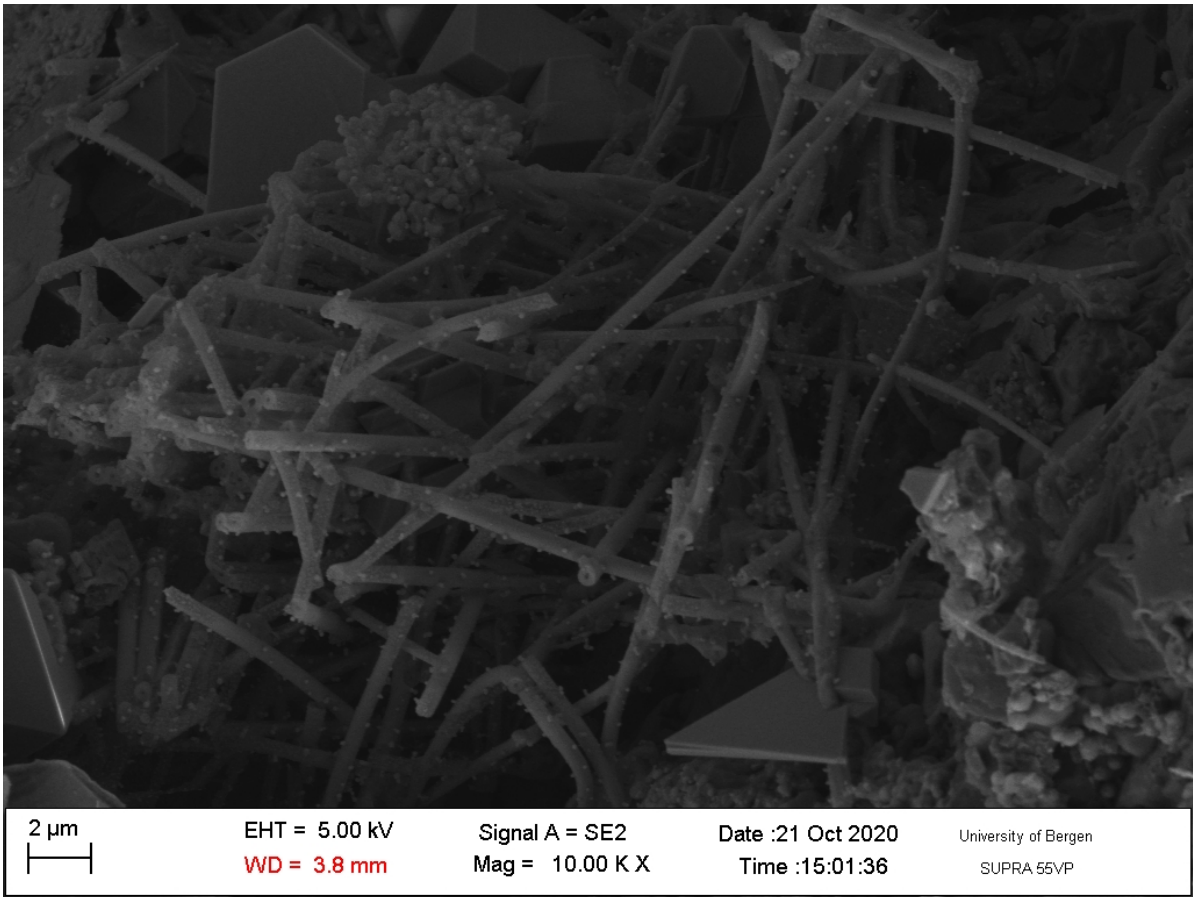
2 µm
EHT = 5.00 kV
WD = 3.8 mm
Signal A = SE2
Mag = 10.00 K X
Date :21 Oct 2020
Time :15:01:36
University of Bergen
SUPRA 55VP

Supplementary material 4: List of primer sets

Table 1: List of primers successfully used in this study.

Gene	Use	Primers	Sequences 5'-3'	PCR cycle	Reference
16S rRNA - Uni	Sequencing	519f	CAGCMGCCGCGGTAA	94°C 30s; 56°C 30s; 72°C 30s	Zhao *et al.* (2019)
		805r	GACTACHVGGGTATCTAATCC		
16S rRNA - Uni	Sequencing	515F	GTGCCAGCMGCCGCGGTAA	94°C 45s, 50°C 60s, 72°C 90s	Caporaso *et al.* (2011)
		806R	GGACTACHVGGGTWTCTAAT		
16S rRNA - Arc	qPCR	519f	CAGCMGCCGCGGTAA	95°C 15s, 60°C 30 s, 72°C 45 s	Jørgensen *et al.* (2012)
		Arc908r	CCCGCCAATTCCTTTAAGTT		
16S rRNA - Bac	qPCR	bac341f	CCTACGGGWGGCWGCA	95°C 15s, 58°C 30s, 72°C 30s	Zhao *et al.* (2019)
		uni518r	ATTACCGCGGCTGCTGG		
aprA	qPCR	aprA-1-FW	TGGCAGATCATGATYMAYGG	94°C 45s, 60°C 45s, 72°C 60s	Meyer and Kuever (2007)
		aprA-5R	GCGCCAACYGGRCCRTA		
dsrA	qPCR	DSR1-F+	ACSCACTGGAAGCACGGCGG	95°C 15s, 59°C 30s, 72°C 30s, 75°C 10s	Spence *et al.* (2008)
		DSR-R	GTGGMRCCGTGCAKRTTGG		
soxB	qPCR	soxB710F	ATCGGYCAGGCYTTYCCSTA	94°C 60s, 50°C 45s 72°C 75s	Leleika *et al.* (2018)
		soxB1184R	MAVGGCCCGTTGAARTTGC		
arrA	qPCR	ArrA-CVF1	CACAGCGCCATCTGCGCCGA	94°C 45s, 60°C 45s, 72°C 60s	Mirza *et al.* (2016)
		ArrA-CVR1	CCGACGAACTCCYTGYTCCA		
aoxB	qPCR	M1-2F	CCACTTCTGCATCGTGGGNTGY GGNTA	95°C 15s, 60°C 45s, 72°C 30s	Hu *et al.* (2015)
		M2-1R	GGAGTTGTAGGCGGGCCKRTTR TGDAT		
nirK	qPCR	nirK_F1aCu	ATCATGGTSCTGCCGCG	95°C 30s, 56°C 45s, 72°C 45s, 80°C 20s	Throbäck *et al.* (2004)
		nirK_R3Cu	GCCTCGATCAGRTTGTGGTT		
nirS	qPCR	Cd3aF	GTSAACGTSAAGGARACSGG	95°C 15s, 51°C 30s, 72°C 45s	Throbäck *et al.* (2004)
		R3cd	GASTTCGGRTGSGTCTTGA		
mcrA	qPCR	MLf	GGTGGTGTMGGATTCACACART AYGCWACAGC	95°C 60s, 49°C 60s, 72°C 180s	Morris *et al.* (2014)
		MLr	TTCATTGCRTAGTTWGGRTAGT T		

Table 2: List of primers tested but not used in the final study. In green, no PCR amplification was shown on a subset of samples (C13 at 2 and 12 cm depth, C16, C27 and C28 at 2 and 10 cm depth) but a positive control was used. The genes are therefore expected not to be detected in our samples. In red, no PCR amplification was shown on the same subset of samples, but no positive control was used, and we can therefore not take any conclusion on the presence or absence of the genes. In blue, the genes were detected through qPCR, but the resulting amplification patterns were abnormal, mainly due to low efficiency, and the results were therefore not used here.

Gene	Use	Primers	Sequences 5'-3'	PCR program	Reference
hzo	qPCR	hzo_F1	TGTGCATGGTCAATTGAAAG	95°C 60s, 53°C 60s, 72°C 120s	Li *et al.* (2010)
		hzo_R1	CAACCTCTTCWGCAGGTGCATG		
amoA - Arc	qPCR	CrenamoA23f	ATGGTCTGGCTWAGACG	95°C 30s, 50°C 45s, 72°C 45s	Tourna *et al.* (2008)
		CrenamoA616r	GCCATCCATCTGTATGTCCA		
amoA - Bac	qPCR	AmoA1f	GGGGTTTCTACTGGTGGT	94°C 15s, 55°C 45s, 72°C 60s	Rotthauwe *et al.* (1997)
		AmoA2r	CCCCTCKGAGCCTTCTTC		
arsC	qPCR	arsC2F	GCGTACAGGCGAAGATGAATA	95°C 15s, 60°C 60s, 72 90s	Jesser *et al.* (2015)

		arsC2R	ACAACAACAGGACGTCAA		
arxA	qPCR	arxA-01F	CTCAGCAGTCTCTATGGTTC	95°C 30s, 58°C 30s, 72°C 30s	Zhao *et al.* (2018)
		arxA-01R	GTGTTAGCGTAGTCGTAGG		
arsB	qPCR	6 different primer sets, see reference			Zhao *et al.* (2018)
psbA	qPCR	psbA-1F	TAYCCNATYTGGGAAGC	94°C 60s, 55°C 60s, 72°C 60s	Man-Aharonovich *et al.* (2010)
		psbA-2R	TCRAGDGGGAARTTRTG		
nifH	qPCR	nifHfw	GGHAARGGHGGHATHGGNAARTC	94°C 30s, 55°C 30s, 72°C 60s	Mehta *et al.* (2003)
		nifHrv	GGCATNGCRAANCCVCCRCANAC		
pmoA	qPCR	A189F	GGNGACTGGGACTTCTGG	94°C 60s, 56°C 60s, 72°C 60s	Bourne *et al.* (2001)
		A650R	ACGTCCTTACCGAAGGT		
soxB	qPCR	soxB693F	ATCGGNCARGCNTTYCCNTA	95°C 30s, 55°C 40s, 72°C 60s	Zhang *et al.* (2017)
		soxB1446B	CATGTCNCCNCCRTGYTG		
soxB	qPCR	soxB693F	ATCGGNCARGCNTTYCCNTA	95°C 30s, 55°C 40s, 72°C 45s	Krishnani *et al.* (2010)
		soxB1164B145	AAGTTGCCDCGNCGRTA		

Bourne, D.G., McDonald, I.R., and Murrell, J.C. (2001) Comparison of pmoA PCR Primer Sets as Tools for Investigating Methanotroph Diversity in Three Danish Soils. Appl Environ Microbiol 67: 3802–3809.

Caporaso, J.G., Lauber, C.L., Walters, W.A., Berg-Lyons, D., Lozupone, C.A., Turnbaugh, P.J., Fierer, N., Knight, R. (2011) Global patterns of 16S rRNA diversity at a depth of millions of sequences per sample. PNAS doi.org/10.1073/pnas.1000080107

Hu, M., Li, F., Liu, C., & Wu, W. (2015). The diversity and abundance of As (III) oxidizers on root iron plaque is critical for arsenic bioavailability to rice. Scientific reports, 5, 13611

Jesser, K. J., Fullerton, H., Hager, K. W., & Moyer, C. L. (2015). Quantitative PCR analysis of functional genes in iron-rich microbial mats at an active hydrothermal vent system (Lō'ihi Seamount, Hawai'i). Applied and environmental microbiology, 81(9), 2976-2984

Jørgensen, S.L., Hannisdal, B., Lanzén, A., Baumberger, T., Flesland, K., Fonseca, R., et al. (2012) Correlating microbial community profiles with geochemical data in highly stratified sediments from the Arctic Mid-Ocean Ridge. Proc Natl Acad Sci U S A 109: E2846-2855

Krishnani, K. K., Kathiravan, V., Natarajan, M., Kailasam, M., & Pillai, S. M. (2010). Diversity of sulfur-oxidizing bacteria in greenwater system of coastal aquaculture. Applied biochemistry and biotechnology, 162(5), 1225-1237

Leleika, S, Harmon, A, Eisenlord, S (2018) Quantifying Sulfur oxidizing Bacteria using qPCR. Corrosion conference and expo 2018

Li, M., Hong, Y., Klotz, M.G., and Gu, J.-D. (2010) A comparison of primer sets for detecting 16S rRNA and hydrazine oxidoreductase genes of anaerobic ammonium-oxidizing bacteria in marine sediments. Appl Microbiol Biotechnol 86: 781–790

Man-Aharonovich, D., Philosof, A., Kirkup, B. C., Le Gall, F., Yogev, T., Berman-Frank, I., ... & Béja, O. (2010). Diversity of active marine picoeukaryotes in the Eastern Mediterranean Sea unveiled using photosystem-II psbA transcripts. The ISME journal, 4(8), 1044-1052

Mehta, M.P., Butterfield, D.A., and Baross, J.A. (2003) Phylogenetic diversity of nitrogenase (nifH) genes in deep-sea and hydrothermal vent environments of the Juan de Fuca Ridge. Appl Environ Microbiol 69: 960–970.

Meyer, B. and Kuever, J. (2007) Molecular Analysis of the Diversity of Sulfate-Reducing and Sulfur-Oxidizing Prokaryotes in the Environment, Using aprA as Functional Marker Gene. Appl Environ Microbiol 73: 7664–7679

Mirza, B. S., Sorensen, D. L., Dupont, R. R., & McLean, J. E. (2017). New arsenate reductase gene (arrA) PCR primers for diversity assessment and quantification in environmental samples. Applied and environmental microbiology, 83(4)

Morris, R., Schauer-Gimenez, A., Bhattad, U., Kearney, C., Struble, C.A., Zitomer, D., and Maki, J.S. (2014) Methyl coenzyme M reductase (mcrA) gene abundance correlates with activity measurements of methanogenic H2/CO2-enriched anaerobic biomass. Microb Biotechnol 7: 77–84

Rotthauwe, J.H., Witzel, K.P., and Liesack, W. (1997) The ammonia monooxygenase structural gene amoA as a functional marker: molecular fine-scale analysis of natural ammonia-oxidizing populations. Appl Environ Microbiol 63: 4704–4712.

Spence, C., Whitehead, T.R., and Cotta, M.A. (2008) Development and comparison of SYBR Green quantitative real-time PCR assays for detection and enumeration of sulfate-reducing bacteria in stored swine manure. J Appl Microbiol 105: 2143–2152

Throbäck, I.N., Enwall, K., Jarvis, Å., and Hallin, S. (2004) Reassessing PCR primers targeting nirS, nirK and nosZ genes for community surveys of denitrifying bacteria with DGGE. FEMS Microbiol Ecol 49: 401–417

Tourna, M., Freitag, T.E., Nicol, G.W., and Prosser, J.I. (2008) Growth, activity and temperature responses of ammonia-oxidizing archaea and bacteria in soil microcosms. Environ Microbiol 10: 1357–1364

Zhang, Y., Wang, X., Zhen, Y., Mi, T., He, H., & Yu, Z. (2017). Microbial diversity and community structure of sulfate-reducing and sulfur-oxidizing bacteria in sediment cores from the east china sea. Frontiers in Microbiology, 8, 2133

Zhao, Y., Su, J. Q., Ye, J., Rensing, C., Tardif, S., Zhu, Y. G., & Brandt, K. K. (2018). AsChip: a high-throughput qPCR chip for comprehensive profiling of genes linked to microbial cycling of arsenic. Environmental science & technology, 53(2), 798-807

Zhao, R., Hannisdal, B., Mogollon, J.M., Jørgensen, S.L. (2019) Nitrifier abundance and diversity peak at deep redox transition zones *Scientific Reports* doi.org/10.1038/s41598-019-44585-6

Supplementary material 5: Other PCA plots

Here are presented several CoDa-PCA form biplots using different taxonomic levels, as well as the biplot of PC1 vs PC3 complement of **Figure 2A**.

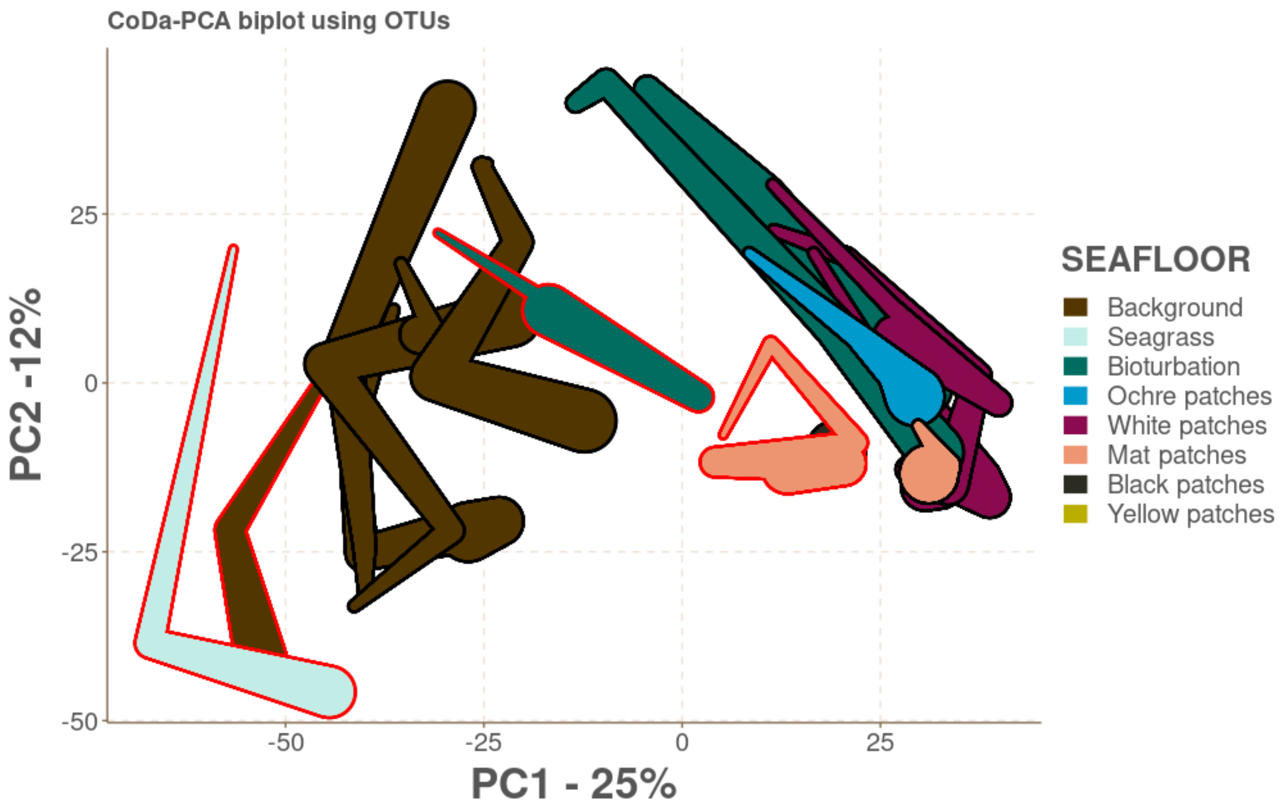

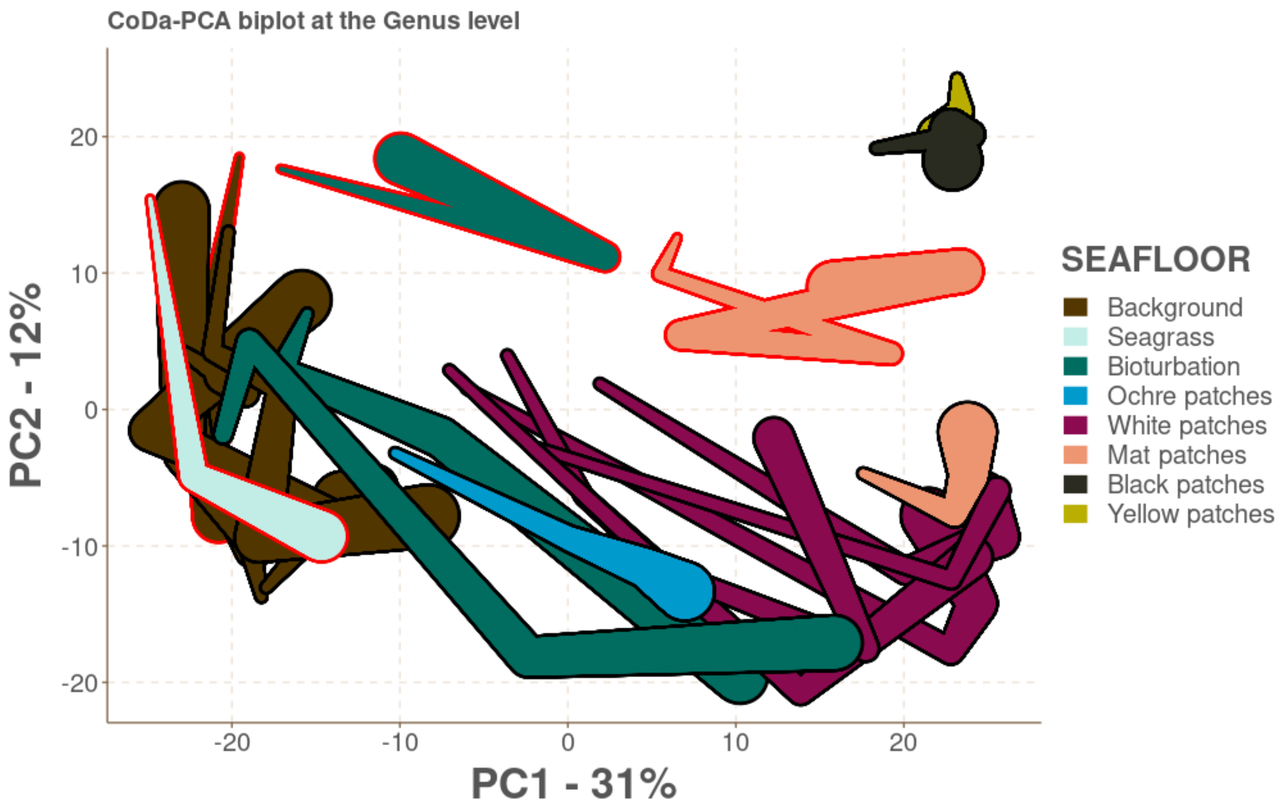

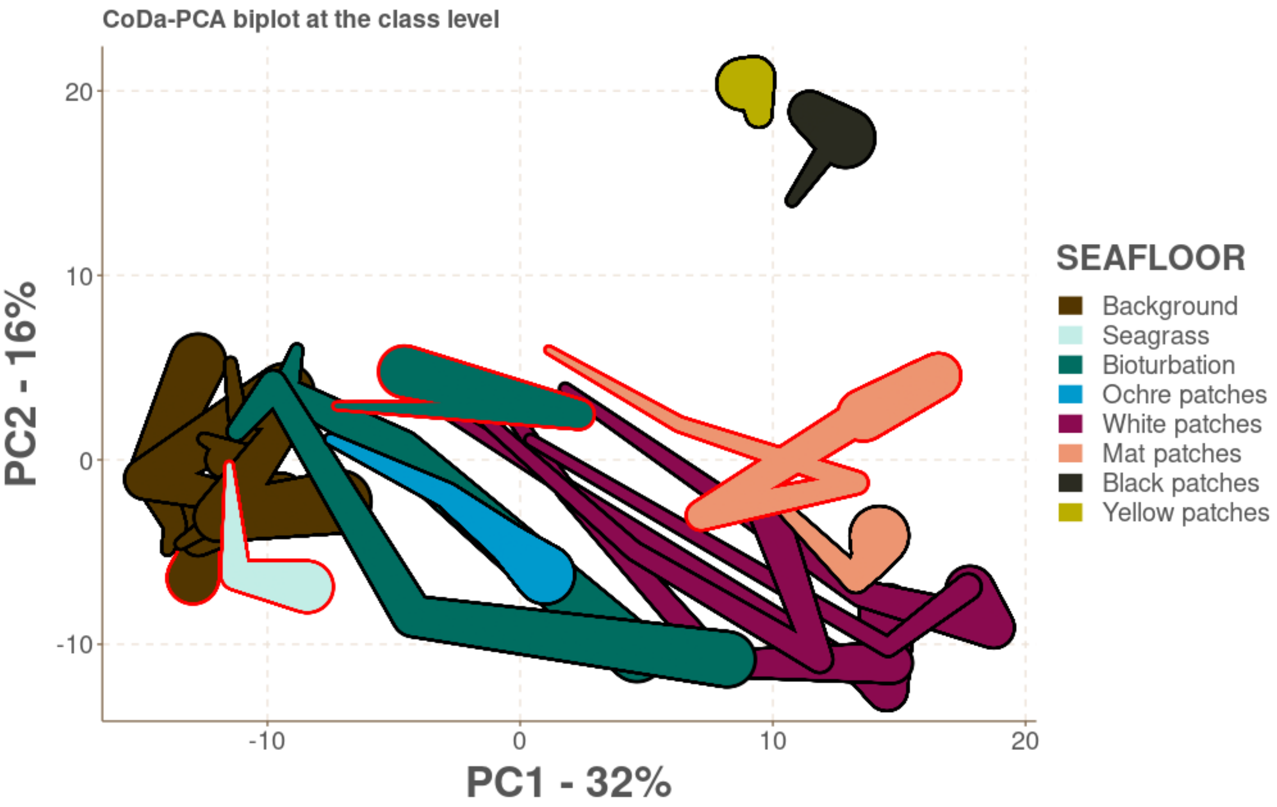
CoDa-PCA biplot at the class level
PC2 - 16%
PC1 - 32%
20
10
0
-10
-10
0
10
20
SEAFLOOR
Background
Seagrass
Bioturbation
Ochre patches
White patches
Mat patches
Black patches
Yellow patches

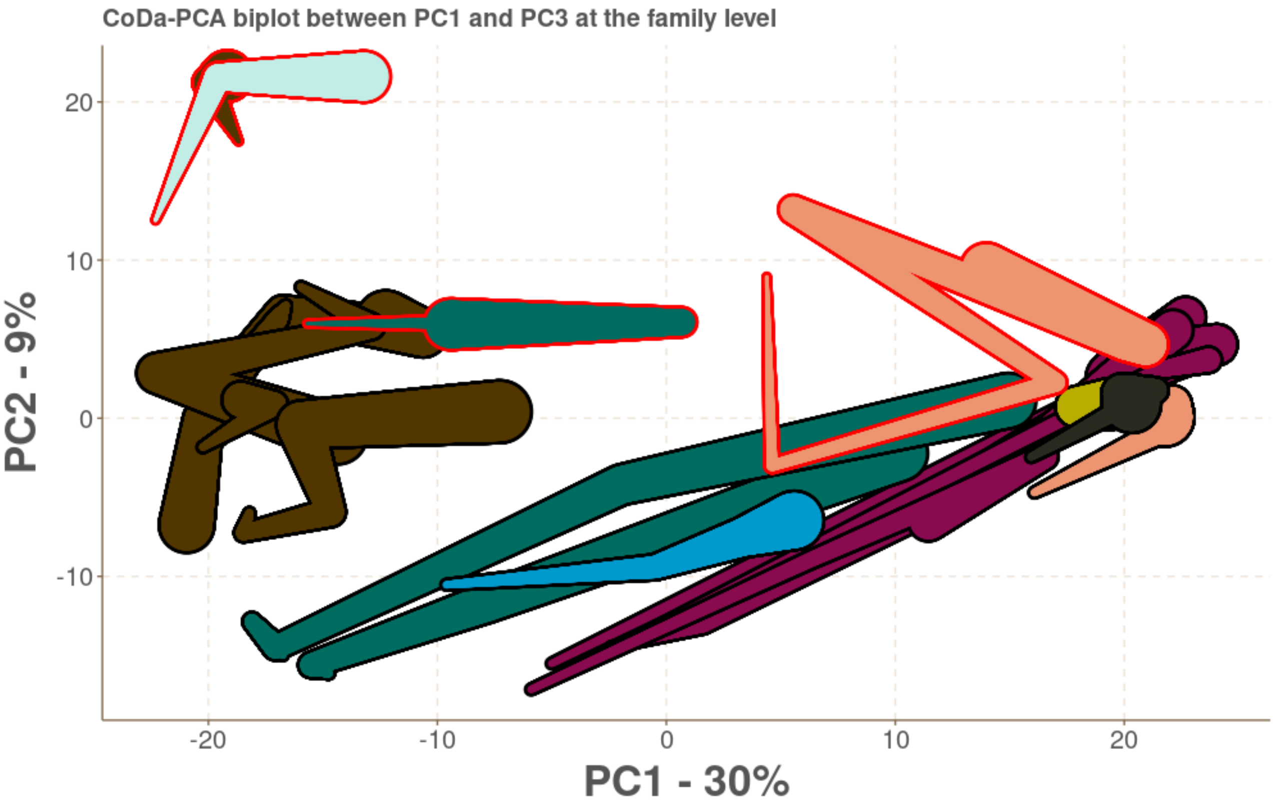
CoDa-PCA biplot between PC1 and PC3 at the family level
PC2 - 9%
PC1 - 30%
20
10
0
-10
-20
-10
0
10
20

Supplementary material 6: Primer *in silico* analysis

519F/805R vs. 515F/806R

The 16S rRNA primer sets used in this study differ between the two datasets. While the Saganaki dataset is produced using the highly used 515F/806R described by Caporaso *et al* (2011), the CarDHynAl dataset is produced using the 519F/805R primers. While being different, both primer sets amplify the same V4/V5 hyper-variable regions, making it easy to analyse them together. We however observe a segregation of the Saganaki samples from the CarDHynAl samples on the PC analysis (**Main article Figure 2**, **Supplementary material 5**), mainly on PC2 and PC3. While the observation that temperature (PC1) remains the main structuring factor for both dataset strengthens this claim, we performed an *in silico* analysis using the RDP probe match tool (http://rdp.cme.msu.edu/probematch/search.jsp) to estimate if the differences observed are linked to primer set specificity or to biological heterogeneity. The following results describe the *in silico* analysis allowing for zero mismatch, except when specified otherwise.

The results in the **Table 1** below shows that the primer sets are very similar when it comes to specificity within the Bacteria domain, targeting each slightly above 2 million targets over the 3.2 million available in the database. Within the domain, they show the same lack of specificity for the *Caldiserica, Chlamydiae, Chlorobi, Parcubacteria, Microgenomates, Candidatus Saccharibacteria, Poribacteria, Cloacimonetes, Candidatus Calescamantes* phyla (in red in **Table 1**). The main difference being the much weaker affinity for the *Chloroflexi* phylum of the 519F/805R set compared to the 515F/806R (in blue in **Table 1**). We also looked specifically at the *Campilobacterota* phylum, as its members show high relative abundances in our study. Once more there is little difference between both primer sets (**Table 2**). However, both primer sets will consistently have a rather low coverage of the families detected in the study (*Arcobacteraceae, Campylobacteraceae, Sulfurovaceae, Thiovulaceae*/*Sulfurimonadaceae*), suggesting that the fraction of the community represented by these families in our samples may be underestimated.

The results in **Table 3** show that the primer set 519F/805R performs better within the Archaea domain, with around 121 thousands over 160 thousands matches, while the 515F/806R matches only around 70 thousands. While both primer sets have very low coverage of the *Nanoarchaeota* and *Nanohaloarchaeota* phyla (in red in **Table 3**), the main difference is the low coverage of *Crenarchaeota, Thaumarchaeota/Nitrososphaeraeota* and *Korarchaeota* phyla by the 515F/806R primer set (in blue in **Table 3**). However, the difference is highly compensated when allowing for one mismatch, as the 519F/805R and 515F/806R primer sets are matching 132 and 126 thousand targets, respectively. The general higher coverage of Archaea by the 519F/805R primer set suggests that the lower relative abundance of Archaea in the CarDHynAl dataset compared to the Saganaki dataset is likely due to real biological heterogeneity.

Table 1: *In silico* analysis of the 16S rRNA primer sets used in this study. Blast again the Bacteria domain allowing for 0 mismatch.

**519F/805R**	**515F/806R**
domain Bacteria (2029975/3195888)	domain Bacteria (2009261/3195888)
phylum Actinobacteria (208839/363132)	phylum Actinobacteria (206907/363132)

phylum Aquificae (1368/2109)	phylum Aquificae (1348/2109)
phylum Bacteroidetes (252278/388293)	phylum Bacteroidetes (247270/388293)
phylum Caldiserica (1/60)	phylum Caldiserica (1/60)
phylum Chlamydiae (15/1620)	phylum Chlamydiae (5/1620)
phylum Chlorobi (7/1549)	phylum Chlorobi (7/1549)
phylum Chloroflexi (12662/44858)	phylum Chloroflexi (20055/44858)
phylum Chrysiogenetes (13/13)	phylum Chrysiogenetes (13/13)
phylum Deferribacteres (1783/2330)	phylum Deferribacteres (1732/2330)
phylum Deinococcus-Thermus (3583/5089)	phylum Deinococcus-Thermus (3548/5089)
phylum Dictyoglomi (45/70)	phylum Dictyoglomi (42/70)
phylum Elusimicrobia (621/798)	phylum Elusimicrobia (620/798)
phylum Fibrobacteres (3198/4160)	phylum Fibrobacteres (3143/4160)
phylum Fusobacteria (13890/17999)	phylum Fusobacteria (13792/17999)
phylum Gemmatimonadetes (4796/7780)	phylum Gemmatimonadetes (4726/7780)
phylum Lentisphaerae (1825/2170)	phylum Lentisphaerae (1760/2170)
phylum Nitrospirae (3623/7937)	phylum Nitrospirae (3552/7937)
phylum Planctomycetes (24444/41117)	phylum Planctomycetes (22925/41117)
phylum Proteobacteria (680897/1065544)	phylum Proteobacteria (672370/1065544)
phylum Spirochaetes (11753/31493)	phylum Spirochaetes (10648/31493)
phylum Synergistetes (2317/3307)	phylum Synergistetes (2285/3307)
phylum Tenericutes (6592/11037)	phylum Tenericutes (6400/11037)
phylum Thermodesulfobacteria (209/250)	phylum Thermodesulfobacteria (206/250)
phylum Thermotogae (1131/1527)	phylum Thermotogae (1117/1527)
phylum BRC1 (506/631)	phylum BRC1 (505/631)
phylum Parcubacteria (2/988)	phylum Parcubacteria (0/988)
phylum Microgenomates (1/335)	phylum Microgenomates (0/335)
phylum SR1 (917/1092)	phylum SR1 (905/1092)
phylum Candidatus Saccharibacteria (27/7867)	phylum Candidatus Saccharibacteria (22/7867)
phylum Latescibacteria (784/1204)	phylum Latescibacteria (769/1204)
phylum Armatimonadetes (1963/3694)	phylum Armatimonadetes (1936/3694)
phylum Verrucomicrobia (15368/28576)	phylum Verrucomicrobia (15069/28576)
phylum Acidobacteria (42366/79741)	phylum Acidobacteria (41784/79741)
phylum Firmicutes (609419/838136)	phylum Firmicutes (606962/838136)
phylum Cyanobacteria/Chloroplast (49559/73370)	phylum Cyanobacteria/Chloroplast (45570/73370)
phylum Marinimicrobia (1124/1682)	phylum Marinimicrobia (1103/1682)
phylum Aminicenantes (2278/3121)	phylum Aminicenantes (2233/3121)
phylum Omnitrophica (30/40)	phylum Omnitrophica (29/40)
phylum Acetothermia (55/87)	phylum Acetothermia (52/87)
phylum Poribacteria (59/363)	phylum Poribacteria (52/363)
phylum Atribacteria (129/171)	phylum Atribacteria (129/171)
phylum Cloacimonetes (691/11489)	phylum Cloacimonetes (694/11489)

phylum Candidatus Calescamantes (0/5)	phylum Candidatus Calescamantes (0/5)
phylum candidate division WPS-1 (718/1533)	phylum candidate division WPS-1 (711/1533)
phylum candidate division WPS-2 (457/813)	phylum candidate division WPS-2 (434/813)
phylum Hydrogenedentes (614/1066)	phylum Hydrogenedentes (611/1066)
phylum candidate division ZB3 (87/94)	phylum candidate division ZB3 (87/94)
phylum Ignavibacteriae (1400/2187)	phylum Ignavibacteriae (1384/2187)
phylum Nitrospinae (466/913)	phylum Nitrospinae (460/913)
phylum Balneolaeota (700/1034)	phylum Balneolaeota (687/1034)
phylum Rhodothermaeota (4256/5162)	phylum Rhodothermaeota (4230/5162)
phylum Kiritimatiellaeota (604/1197)	phylum Kiritimatiellaeota (589/1197)
phylum Campilobacterota (14453/22069)	phylum Campilobacterota (14302/22069)
phylum Abditibacteriota (347/655)	phylum Abditibacteriota (104/655)
phylum Coprothermobacterota (353/474)	phylum Coprothermobacterota (349/474)

Table 2: *In silico* analysis of the 16S rRNA primer sets used in this study. Blast again the *Campylobacterota* phylum allowing for 0 mismatch.

519F/805R	515F/806R
phylum Campilobacterota (14453/22069)	phylum Campilobacterota (14302/22069)
class Campylobacteria (14353/21937)	class Campylobacteria (14209/21937)
order Campylobacterales (14112/21544)	order Campylobacterales (13968/21544)
family Campylobacteraceae (2367/3263)	family Campylobacteraceae (2353/3263)
genus Campylobacter (2367/3263)	genus Campylobacter (2353/3263)
unclassified_Campylobacteraceae (0/0)	unclassified_Campylobacteraceae (0/0)
family Helicobacteraceae (1602/1849)	family Helicobacteraceae (1597/1849)
genus Helicobacter (1549/1760)	genus Helicobacter (1549/1760)
genus Wolinella (43/57)	genus Wolinella (38/57)
unclassified_Helicobacteraceae (10/32)	unclassified_Helicobacteraceae (10/32)
family Hydrogenimonaceae (57/98)	family Hydrogenimonaceae (55/98)
genus Hydrogenimonas (57/98)	genus Hydrogenimonas (55/98)
unclassified_Hydrogenimonaceae (0/0)	unclassified_Hydrogenimonaceae (0/0)
family Arcobacteraceae (2878/4737)	family Arcobacteraceae (2839/4737)
genus Arcobacter (112/152)	genus Arcobacter (110/152)
genus Aliarcobacter (438/604)	genus Aliarcobacter (428/604)
genus Pseudarcobacter (486/699)	genus Pseudarcobacter (478/699)
genus Poseidonibacter (404/719)	genus Poseidonibacter (394/719)
genus Malaciobacter (209/262)	genus Malaciobacter (209/262)
genus Halarcobacter (117/152)	genus Halarcobacter (117/152)
unclassified_Arcobacteraceae (1112/2149)	unclassified_Arcobacteraceae (1103/2149)
family Nitratiruptoraceae (37/52)	family Nitratiruptoraceae (37/52)
genus Nitratiruptor (37/52)	genus Nitratiruptor (37/52)

unclassified_Nitratiruptoraceae (0/0)	unclassified_Nitratiruptoraceae (0/0)
family Sulfurospirillaceae (784/1103)	family Sulfurospirillaceae (783/1103)
genus Sulfurospirillum (784/1103)	genus Sulfurospirillum (783/1103)
unclassified_Sulfurospirillaceae (0/0)	unclassified_Sulfurospirillaceae (0/0)
family Sulfurovaceae (3493/5595)	family Sulfurovaceae (3442/5595)
genus Sulfurovum (3285/5310)	genus Sulfurovum (3238/5310)
genus Nitratifractor (105/140)	genus Nitratifractor (106/140)
unclassified_Sulfurovaceae (103/145)	unclassified_Sulfurovaceae (98/145)
family Thiovulaceae (2287/3684)	family Thiovulaceae (2269/3684)
genus Thiovulum (47/52)	genus Thiovulum (47/52)
genus Sulfurimonas (1448/2287)	genus Sulfurimonas (1435/2287)
genus Sulfuricurvum (672/1163)	genus Sulfuricurvum (669/1163)
unclassified_Thiovulaceae (120/182)	unclassified_Thiovulaceae (118/182)
unclassified_Campylobacterales (607/1163)	unclassified_Campylobacterales (593/1163)
order Nautiliales (223/331)	order Nautiliales (223/331)
family Nautiliaceae (168/231)	family Nautiliaceae (168/231)
genus Caminibacter (61/96)	genus Caminibacter (61/96)
genus Lebetimonas (16/16)	genus Lebetimonas (16/16)
genus Nautilia (27/34)	genus Nautilia (27/34)
genus Cetia (9/10)	genus Cetia (9/10)
unclassified_Nautiliaceae (55/75)	unclassified_Nautiliaceae (55/75)
family Thioreductoraceae (55/96)	family Thioreductoraceae (55/96)
genus Thioreductor (55/96)	genus Thioreductor (55/96)
unclassified_Thioreductoraceae (0/0)	unclassified_Thioreductoraceae (0/0)
unclassified_Nautiliales (0/4)	unclassified_Nautiliales (0/4)
unclassified_Campylobacteria (18/62)	unclassified_Campylobacteria (18/62)
class Desulfurellia (100/132)	class Desulfurellia (93/132)
order Desulfurellales (100/132)	order Desulfurellales (93/132)
family Desulfurellaceae (75/104)	family Desulfurellaceae (68/104)
genus Desulfurella (75/104)	genus Desulfurella (68/104)
unclassified_Desulfurellaceae (0/0)	unclassified_Desulfurellaceae (0/0)
family Hippeaceae (25/28)	family Hippeaceae (25/28)
genus Hippea (25/28)	genus Hippea (25/28)
unclassified_Hippeaceae (0/0)	unclassified_Hippeaceae (0/0)
unclassified_Desulfurellales (0/0)	unclassified_Desulfurellales (0/0)
unclassified_Desulfurellia (0/0)	unclassified_Desulfurellia (0/0)
unclassified_Campilobacterota (0/0)	unclassified_Campilobacterota (0/0)

Table 3: *In silico* analysis of the 16S rRNA primer sets used in this study. Blast again the Archaea domain allowing for 0 mismatch.

519F/805R	515F/806R
domain Archaea (121660/160768)	domain Archaea (70540/160768)
phylum Crenarchaeota (9810/13221)	phylum Crenarchaeota (210/13221)
class Thermoprotei (9810/13221)	class Thermoprotei (210/13221)
unclassified_Crenarchaeota (0/0)	unclassified_Crenarchaeota (0/0)
phylum Euryarchaeota (67444/90164)	phylum Euryarchaeota (62751/90164)
class Archaeoglobi (277/646)	class Archaeoglobi (33/646)
class Halobacteria (8245/10475)	class Halobacteria (8033/10475)
class Methanobacteria (12618/16375)	class Methanobacteria (11852/16375)
class Methanococci (418/612)	class Methanococci (413/612)
class Methanomicrobia (26707/37631)	class Methanomicrobia (24877/37631)
class Methanopyri (19/21)	class Methanopyri (0/21)
class Thermococci (678/1013)	class Thermococci (4/1013)
class Thermoplasmata (10728/13525)	class Thermoplasmata (9918/13525)
unclassified_Euryarchaeota (7754/9866)	unclassified_Euryarchaeota (7621/9866)
phylum Korarchaeota (214/280)	phylum Korarchaeota (70/280)
genus Candidatus Korarchaeum (214/280)	genus Candidatus Korarchaeum (70/280)
unclassified_Korarchaeota (0/0)	unclassified_Korarchaeota (0/0)
phylum Nanoarchaeota (0/195)	phylum Nanoarchaeota (0/195)
phylum Thaumarchaeota (32117/40254)	phylum Thaumarchaeota (94/40254)
order Cenarchaeales (0/0)	order Cenarchaeales (0/0)
order Nitrosopumilales (18875/23925)	order Nitrosopumilales (70/23925)
order Nitrososphaerales (12585/15368)	order Nitrososphaerales (23/15368)
unclassified_Thaumarchaeota (657/961)	unclassified_Thaumarchaeota (1/961)
phylum Nanohaloarchaeota (21/159)	phylum Nanohaloarchaeota (0/159)
class Nanohaloarchaea (0/0)	
genus Candidatus Nanosalina (21/159)	
unclassified_Nanohaloarchaeota (0/0)	
phylum Woesearchaeota (1496/1993)	phylum Woesearchaeota (1448/1993)
genus Woesearchaeota Incertae Sedis AR15 (20/49)	genus Woesearchaeota Incertae Sedis AR15 (19/49)
genus Woesearchaeota Incertae Sedis AR16 (440/598)	genus Woesearchaeota Incertae Sedis AR16 (418/598)
genus Woesearchaeota Incertae Sedis AR17 (1/4)	genus Woesearchaeota Incertae Sedis AR17 (1/4)
genus Woesearchaeota Incertae Sedis AR18 (18/26)	genus Woesearchaeota Incertae Sedis AR18 (18/26)
genus Woesearchaeota Incertae Sedis AR20 (12/17)	genus Woesearchaeota Incertae Sedis AR20 (12/17)
unclassified_Woesearchaeota (1005/1299)	unclassified_Woesearchaeota (980/1299)
phylum Pacearchaeota (1/2)	phylum Pacearchaeota (1/2)
genus Pacearchaeota Incertae Sedis AR13 (1/2)	genus Pacearchaeota Incertae Sedis AR13 (1/2)
unclassified_Pacearchaeota (0/0)	unclassified_Pacearchaeota (0/0)

phylum Aigarchaeota (0/0)	phylum Aigarchaeota (0/0)
phylum Diapherotrites (146/217)	phylum Diapherotrites (0/217)
genus Candidatus Iainarchaeum (0/10)	
genus Diapherotrites Incertae Sedis AR10 (146/200)	
unclassified_Diapherotrites (0/7)	
phylum Aenigmarchaeota (23/34)	phylum Aenigmarchaeota (18/34)
genus Candidatus Aenigmarchaeum (23/34)	genus Candidatus Aenigmarchaeum (18/34)
unclassified_Aenigmarchaeota (0/0)	unclassified_Aenigmarchaeota (0/0)
phylum Parvarchaeota (0/2)	phylum Parvarchaeota (0/2)
Bacteria Outgroup (1/1)	Bacteria Outgroup (1/1)
unclassified_Archaea (10387/14246)	unclassified_Archaea (5947/14246)

soxB710F/soxB1184R

The aim of this *in silico* analysis was to assess how well our *soxB* primers match the genes present in *Arcobacter*, *Sulfurimonas*, and *Sulfurovum* that are known sulfide oxidizers in our samples. We retrieved all the *soxB* sequences assigned to these 3 genera on the fungene database (http://fungene.cme.msu.edu). This resulted in 23 *Arcobacter* sequences, 6 *Sulfurovum* sequences, and 2 *Sulfurimonas* sequences. Then we tested our primers on these 31 sequences, which showed that:

- *soxB710F* has a minimum of 2 mismatches, with the sequence from *Sulfurimonas autotrophica*, but most of the time 4 mismatches with all other sequences. See figure below.

:: Probe Match on soxB Nucleotide :: ATCGGYCAGGCYTTYCCSTA

Edit distance: 0 Probe: 5'ATCGGYCAGGCYTTYCCSTA 3' Target: 5'TASGGRAARGCCTGRCCGAT 3' Total hits ≤ 0 Errors: 22

Return to analysis pag

Analyze Selection | Select All

Selection	Detail	Distance	TARGET 5'TASGGRAARGCCTGRCCGAT 3'<br>PROBE 3'ATCGGYCAGGCYTTYCCSTA 5'	Definition	Organism
☐	AKF25445	4	T5'atcggacagtcattcccgtt<br>P3'TAGCCRGTCCGRAARGGSAT	sulfur oxidation protein SoxB	Sulfurovum lithotrophicum
☐	BAF71461	4	T5'atcggacagtcatttccgtt<br>P3'TAGCCRGTCCGRAARGGSAT	sulfur oxidation protein SoxB	Sulfurovum sp. NBC37-1
☐	ADN09044	2	T5'atcggtcagtcttttccgtt<br>P3'TAGCCRGTCCGRAARGGSAT	sulfate thiol esterase SoxB	Sulfurimonas autotrophica DSM 16294
☐	QDF29102	3	T5'ataggacaggctttccctta<br>P3'TAGCCRGTCCGRAARGGSAT	sulfur oxidation protein, thiosulfate hydrolase	Arcobacter anaerophilus
☐	AXH12373	4	T5'attggtcaagcattccctta<br>P3'TAGCCRGTCCGRAARGGSAT	sulfur oxidation protein, thiosulfate hydrolase	Arcobacter bivalviorum
☐	QEZ88825	3	T5'attggtcaagctttccctta<br>P3'TAGCCRGTCCGRAARGGSAT	sulfur oxidation protein, thiosulfate hydrolase	Arcobacter cibarius
☐	QCZ24557	4	T5'attggtcaagcatttccata<br>P3'TAGCCRGTCCGRAARGGSAT	thiosulfohydrolase SoxB	Arcobacter cryaerophilus ATCC 43158
☐	AYJ80344	4	T5'attggtcaagcatttccata<br>P3'TAGCCRGTCCGRAARGGSAT	sulfur oxidation protein, thiosulfate hydrolase	Arcobacter cryaerophilus ATCC 43158
☐	AYJ77900	3	T5'attggtcaagctttccctta<br>P3'TAGCCRGTCCGRAARGGSAT	sulfur oxidation protein, thiosulfate hydrolase	Arcobacter cryaerophilus D2610
☐	AXX94393	4	T5'attggtcaagcattcccata<br>P3'TAGCCRGTCCGRAARGGSAT	sulfur oxidation protein, thiosulfate hydrolase	Arcobacter ellisii
☐	AXH09029	4	T5'attggacaagcttttcctta<br>P3'TAGCCRGTCCGRAARGGSAT	sulfur oxidation protein, thiosulfate hydrolase	Arcobacter halophilus
☐	AXX86458	4	T5'attggacaagcctttcctta<br>P3'TAGCCRGTCCGRAARGGSAT	sulfur oxidation protein, thiosulfate hydrolase	Arcobacter marinus
☐	AXX91629	4	T5'attggtcaagcatttcctta<br>P3'TAGCCRGTCCGRAARGGSAT	sulfur oxidation protein, thiosulfate hydrolase	Arcobacter molluscorum LMG 25693
☐	ADG93464	4	T5'attggacaagctttccctta<br>P3'TAGCCRGTCCGRAARGGSAT	5'-Nucleotidase domain protein	Arcobacter nitrofigilis DSM 7299
☐	QEP33743	4	T5'attggtcaagcattccctta<br>P3'TAGCCRGTCCGRAARGGSAT	sulfur oxidation protein, thiosulfate hydrolase	Arcobacter pacificus
☐	QEP41225	3	T5'atcggacaagctttcccata<br>P3'TAGCCRGTCCGRAARGGSAT	sulfur oxidation protein, thiosulfate hydrolase	[Arcobacter] porcinus
☐	AZL53446	4	T5'attggacaagctttccctta<br>P3'TAGCCRGTCCGRAARGGSAT	thiosulfohydrolase SoxB	Arcobacter skirrowii
☐	AXX84304	4	T5'attggacaagctttccctta<br>P3'TAGCCRGTCCGRAARGGSAT	sulfur oxidation protein, thiosulfate hydrolase	Arcobacter skirrowii CCUG 10374
☐	BAK72568	3	T5'attggtcaagctttcccata<br>P3'TAGCCRGTCCGRAARGGSAT	sulfur oxidation protein SoxB	Arcobacter sp. L
☐	APW66432	3	T5'attggtcaagctttccctta<br>P3'TAGCCRGTCCGRAARGGSAT	thiosulfohydrolase SoxB	Arcobacter sp. LPB0137
☐	QBF16586	4	T5'atcggacaagcatttccata<br>P3'TAGCCRGTCCGRAARGGSAT	sulfur oxidation protein, thiosulfate hydrolase	Arcobacter thereius LMG 24486
☐	AXK48994	4	T5'attggtcaagcatttccata<br>P3'TAGCCRGTCCGRAARGGSAT	sulfur oxidation protein, thiosulfate hydrolase	Arcobacter trophiarum LMG 25534

- soxB1184R has a minimum of 4 mismatches with the target sequence, and only with *Arcobacter* sequences. Sequences belonging to *Sulforovum* and *Sulfurimonas* have more mismatches. See figure below.

:: Probe Match on soxB Nucleotide :: GCAAYTTCAACGGGCCBTK

Return to analysis page

Edit distance: 0 Probe: 5'GCAAYTTCAACGGGCCBTK 3' Target: 5'MAVGGCCCGTTGAARTTGC 3' Total hits ≤ 0 Errors: 12

Analyze Selection | Select All

Selection	Detail	Distance	TARGET 5'MAVGGCCCGTTGAARTTGC 3'<br>PROBE 3'GCAAYTTCAACGGGCCBTK 5'	Definition	Organism
☐	QDF29102	4	T5'gaaattttaacggttcttg<br>P3'CGTTRAAGTTGCCCGGVAM	sulfur oxidation protein, thiosulfate hydrolase	Arcobacter anaerophilus
☐	AXH12373	4	T5'gtaacttcaatggttcttg<br>P3'CGTTRAAGTTGCCCGGVAM	sulfur oxidation protein, thiosulfate hydrolase	Arcobacter bivalviorum
☐	SNV25538	4	T5'gaaatttcaatggttcttg<br>P3'CGTTRAAGTTGCCCGGVAM	Trifunctional nucleotide phosphoesterase protein YfkN precursor	Arcobacter butzleri
☐	ABV66839	4	T5'gaaatttcaatggttcttg<br>P3'CGTTRAAGTTGCCCGGVAM	sulfur oxidation protein, sulfate thiol esterase	Arcobacter butzleri RM4018
☐	AYJ77900	4	T5'gaaacttcaatggttcttg<br>P3'CGTTRAAGTTGCCCGGVAM	sulfur oxidation protein, thiosulfate hydrolase	Arcobacter cryaerophilus D2610
☐	AXX94393	4	T5'ggaacttcaatggttcttg<br>P3'CGTTRAAGTTGCCCGGVAM	sulfur oxidation protein, thiosulfate hydrolase	Arcobacter ellisii
☐	QEP41225	4	T5'gaaacttcaatggttcttg<br>P3'CGTTRAAGTTGCCCGGVAM	sulfur oxidation protein, thiosulfate hydrolase	[Arcobacter] porcinus
☐	AZL53446	4	T5'gtaacttcaatggttcttg<br>P3'CGTTRAAGTTGCCCGGVAM	thiosulfohydrolase SoxB	Arcobacter skirrowii
☐	AXX84304	4	T5'gtaacttcaatggttcttg<br>P3'CGTTRAAGTTGCCCGGVAM	sulfur oxidation protein, thiosulfate hydrolase	Arcobacter skirrowii CCUG 10374
☐	BAK72568	4	T5'ggaacttcaatggttcttg<br>P3'CGTTRAAGTTGCCCGGVAM	sulfur oxidation protein SoxB	Arcobacter sp. L
☐	QBF16586	4	T5'gaaacttcaatggttcttg<br>P3'CGTTRAAGTTGCCCGGVAM	sulfur oxidation protein, thiosulfate hydrolase	Arcobacter thereius LMG 24486
☐	AXK48994	4	T5'gaaatttcaatggctcttg<br>P3'CGTTRAAGTTGCCCGGVAM	sulfur oxidation protein, thiosulfate hydrolase	Arcobacter trophiarum LMG 25534

This analysis suggests that *soxB* genes belonging to *Campylobacterota* are likely not to be targeted using this primer set. Nevertheless, PCR screening on the microbial mats using these primers produced some amplicons. Therefore, there is possibly a low efficiency amplification happening. Alternatively other SOB than *Campylobacterota* are present in the mat.

Supplementary material 7: Other barplots

Here are presented the barplots at the domain and family taxonomic level. Taxa belonging to the Archaea domain are consistently drawn in shades of red, while taxa belonging to the Bacteria domain are consistently drawn in shades of blue. For the plots at the Family taxonomic level, only the family representing at leats 5% in at least 1 sample are being shown. The others are pooled into the “Others” category.

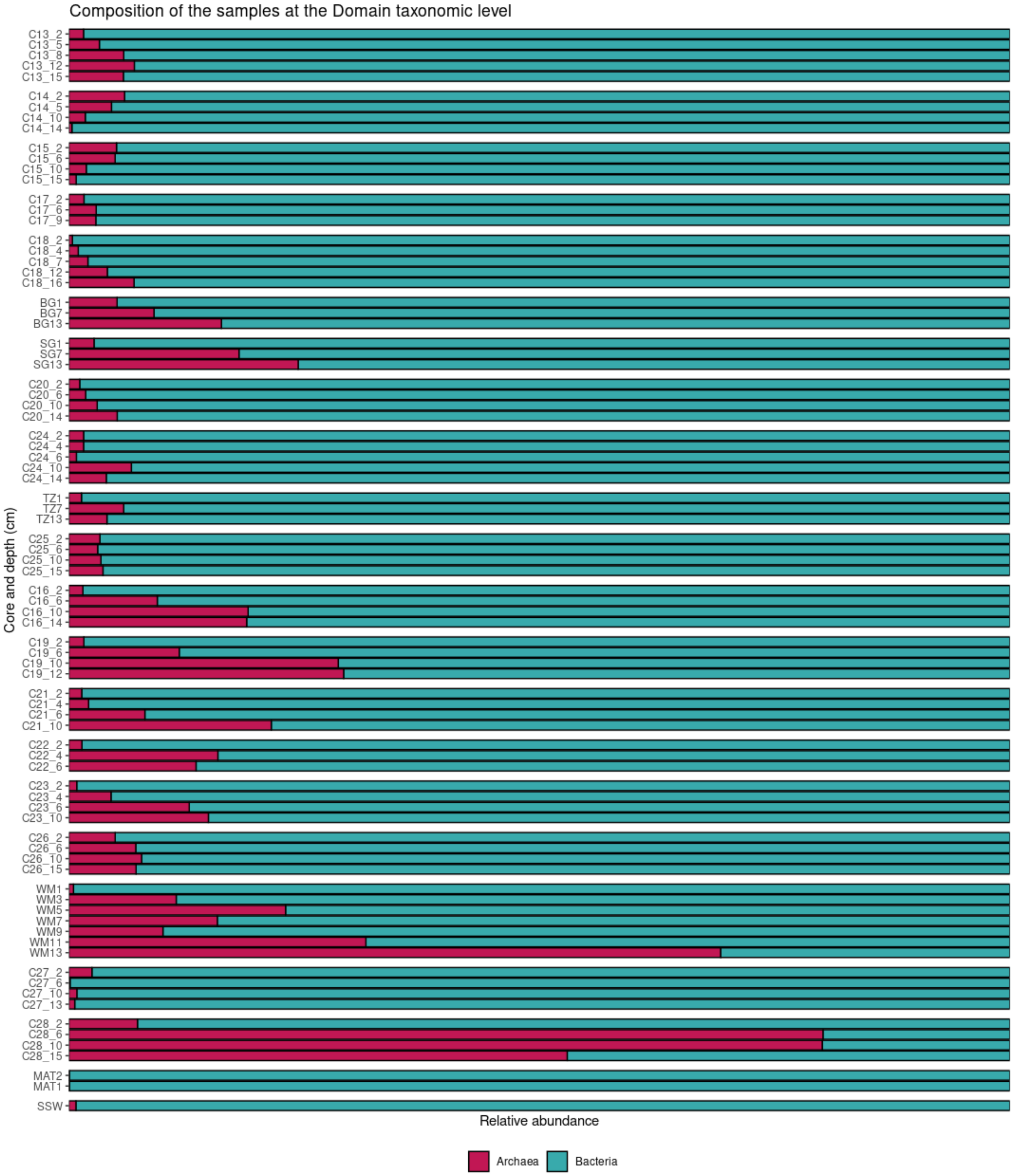

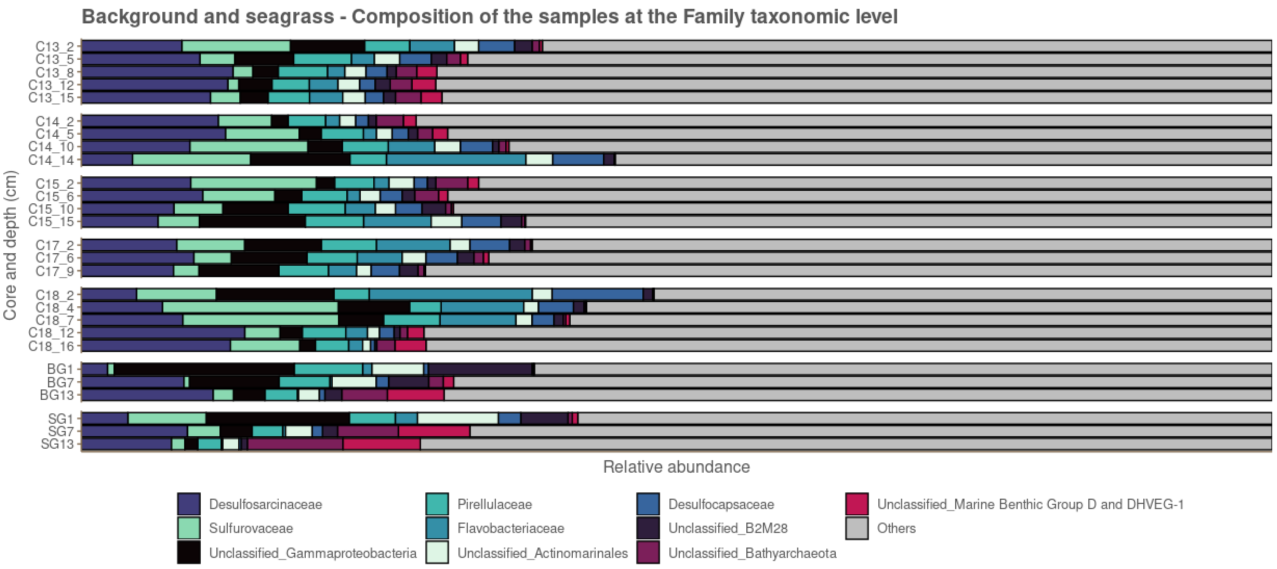
Background and seagrass - Composition of the samples at the Family taxonomic level
C13_2
C13_5
C13_8
C13_12
C13_15
C14_2
C14_5
C14_10
C14_14
C15_2
C15_6
C15_10
C15_15
C17_2
C17_6
C17_9
C18_2
C18_4
C18_7
C18_12
C18_16
BG1
BG7
BG13
SG1
SG7
SG13
Core and depth (cm)
Relative abundance
Desulfosarcinaceae
Sulfurovaceae
Unclassified_Gammaproteobacteria
Pirellulaceae
Flavobacteriaceae
Unclassified_Actinomarinales
Desulfocapsaceae
Unclassified_B2M28
Unclassified_Bathyarchaeota
Unclassified_Marine Benthic Group D and DHVEG-1
Others

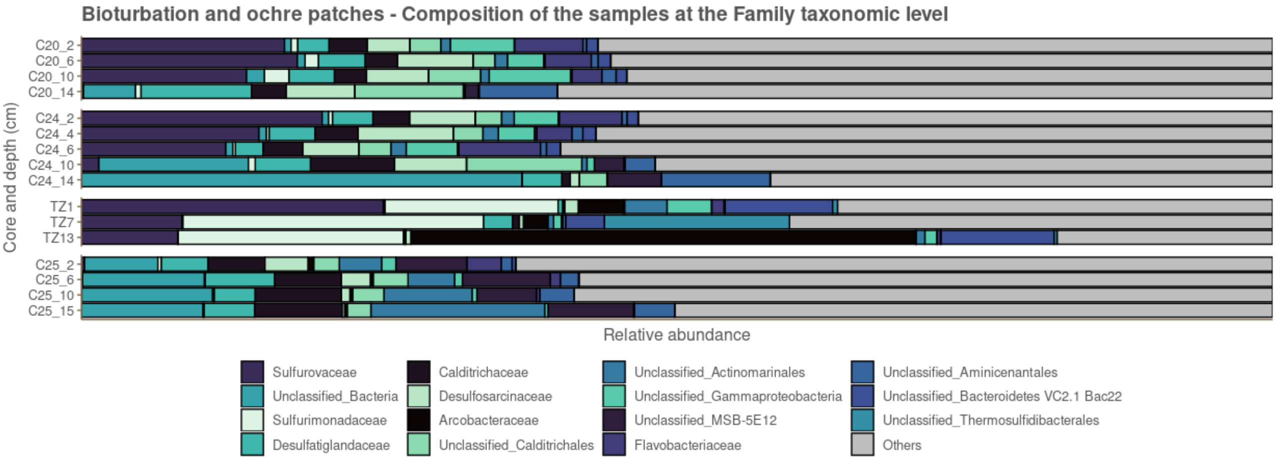
Bioturbation and ochre patches - Composition of the samples at the Family taxonomic level
C20_2
C20_6
C20_10
C20_14
C24_2
C24_4
C24_6
C24_10
C24_14
TZ1
TZ7
TZ13
C25_2
C25_6
C25_10
C25_15
Core and depth (cm)
Relative abundance
Sulfurovaceae
Unclassified_Bacteria
Sulfurimonadaceae
Desulfatiglandaceae
Caldithrichaceae
Desulfosarcinaceae
Arcobacteraceae
Unclassified_Calditrichales
Unclassified_Actinomarinales
Unclassified_Gammaproteobacteria
Unclassified_MSB-5E12
Flavobacteriaceae
Unclassified_Aminicenantales
Unclassified_Bacteroidetes VC2.1 Bac22
Unclassified_Thermosulfidibacterales
Others

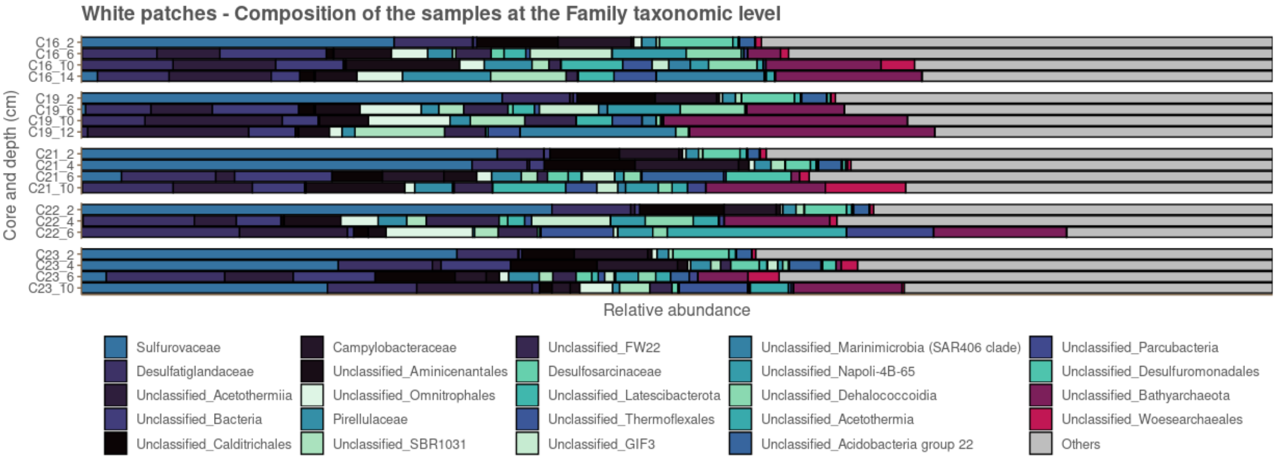
White patches - Composition of the samples at the Family taxonomic level
C16_2
C16_6
C16_10
C16_14
C19_2
C19_6
C19_10
C19_12
C21_2
C21_4
C21_6
C21_10
C22_2
C22_4
C22_6
C23_2
C23_4
C23_6
C23_10
Core and depth (cm)
Relative abundance
Sulfurovaceae
Desulfatiglandaceae
Unclassified_Acetothermiia
Unclassified_Bacteria
Unclassified_Calditrichales
Campylobacteraceae
Unclassified_Aminicenantales
Unclassified_Omnitrophales
Pirellulaceae
Unclassified_SBR1031
Unclassified_FW22
Desulfosarcinaceae
Unclassified_Latescibacterota
Unclassified_Thermoflexales
Unclassified_GIF3
Unclassified_Marinimicrobia (SAR406 clade)
Unclassified_Napoli-4B-65
Unclassified_Dehalococcoidia
Unclassified_Acetothermia
Unclassified_Acidobacteria group 22
Unclassified_Parcubacteria
Unclassified_Desulfuromonadales
Unclassified_Bathyarchaeota
Unclassified_Woesearchaeales
Others

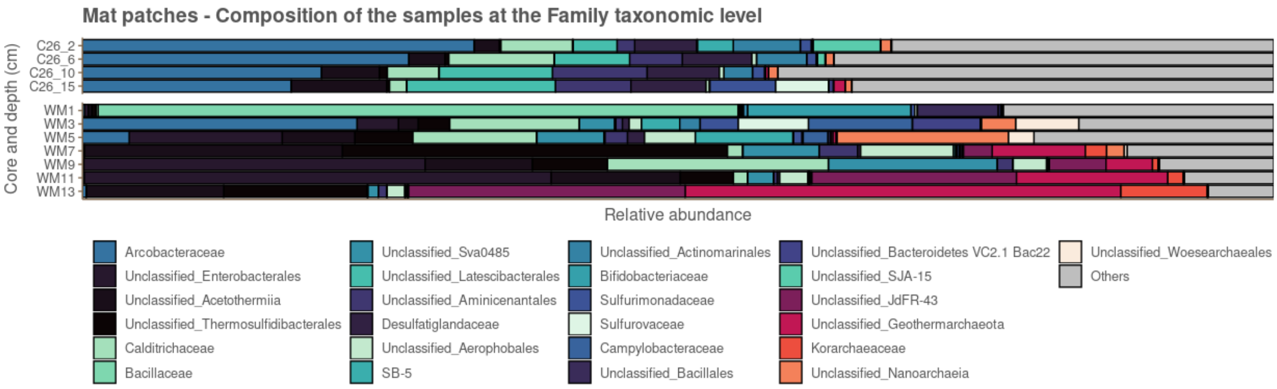
Mat patches - Composition of the samples at the Family taxonomic level
Core and depth (cm)
C26_2
C26_6
C26_10
C26_15
WM1
WM3
WM5
WM7
WM9
WM11
WM13
Relative abundance
Arcobacteraceae
Unclassified_Enterobacterales
Unclassified_Acetothermiia
Unclassified_Thermosulfidibacterales
Calditrichaceae
Bacillaceae
Unclassified_Sva0485
Unclassified_Latescibacterales
Unclassified_Aminicenantales
Desulfatiglandaceae
Unclassified_Aerophobales
SB-5
Unclassified_Actinomarinales
Bifidobacteriaceae
Sulfurimonadaceae
Sulfurovaceae
Campylobacteraceae
Unclassified_Bacillales
Unclassified_Bacteroidetes VC2.1 Bac22
Unclassified_SJA-15
Unclassified_JdFR-43
Unclassified_Geothermarchaeota
Korarchaeaceae
Unclassified_Nanoarchaeia
Unclassified_Woesearchaeales
Others

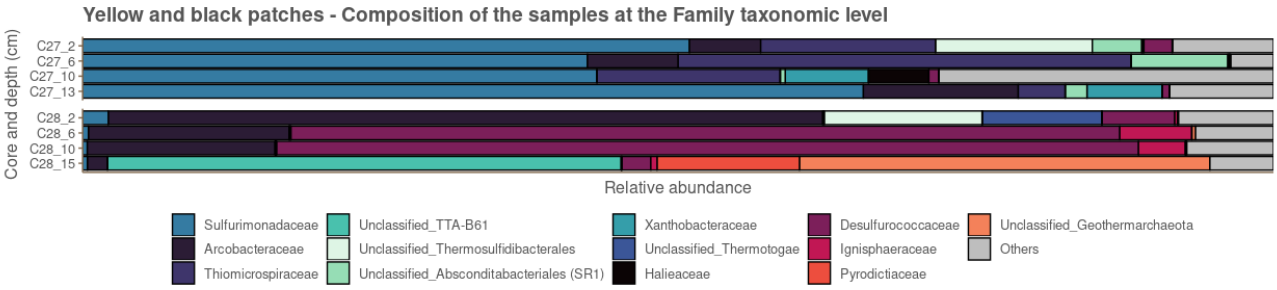
Yellow and black patches - Composition of the samples at the Family taxonomic level
Core and depth (cm)
C27_2
C27_6
C27_10
C27_13
C28_2
C28_6
C28_10
C28_15
Relative abundance
Sulfurimonadaceae
Arcobacteraceae
Thiomicrospiraceae
Unclassified_TTA-B61
Unclassified_Thermosulfidibacterales
Unclassified_Absconditabacteriales (SR1)
Xanthobacteraceae
Unclassified_Thermotogae
Halieaceae
Desulfurococcaceae
Ignisphaeraceae
Pyrodictiaceae
Unclassified_Geothermarchaeota
Others

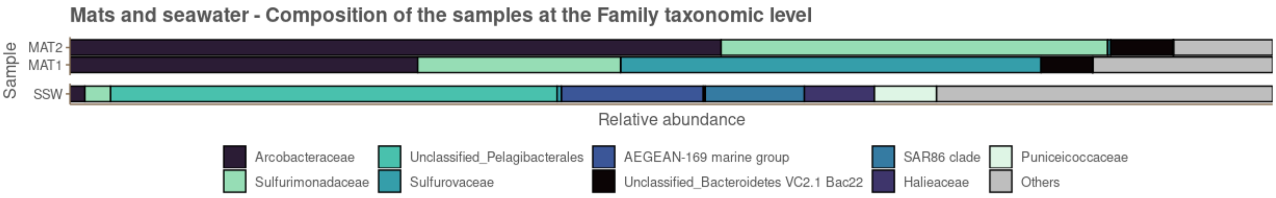
Mats and seawater - Composition of the samples at the Family taxonomic level
Sample
MAT2
MAT1
SSW
Relative abundance
Arcobacteraceae
Sulfurimonadaceae
Unclassified_Pelagibacterales
Sulfurovaceae
AEGEAN-169 marine group
Unclassified_Bacteroidetes VC2.1 Bac22
SAR86 clade
Halieaceae
Puniceicoccaceae
Others

Supplementary material 8: Balances

In microbiology, it is common to try to identify a microbial signature, meaning a group of taxa that can predict an outcome. In our case, this is identifying a group of taxa that would be specific to a seafloor type. In principle, this is the idea behind differential abundance analysis. However, it has been argued that, when using compositional data, the interpretation of changes in single taxa are irrelevant and can lead to false conclusions. Instead, investigating the changes in ratios between groups of taxa has been advocated (Quinn et al. 2021). Balances are one of the approaches that make use of such ratios (See for example Rivera-Pinto *et al.,* 2018).

Here is explained how this is applied to this study.

The formula for a balance is:

$$\sqrt{\frac{(amount\ taxa\ numerator)*(amount\ taxa\ denominator)}{(amount\ taxa\ numerator)+(amount\ taxa\ denominator)}}*\log\frac{((counts\ taxon\ A)*(counts\ taxon\ B)*(...))^{1/taxa\ numerator}}{((counts\ taxon\ C)*(counts\ taxon\ D)*(...))^{1/taxa\ denominator}}$$

In the formula, the numerator and denominator of the log-ratio (right side of the equation) are geometric means which represent the group of taxa. As such, the balance captures the relative change of the group of taxa in the numerator with respect to the group of taxa in the denominator. This means that the balance may change even if both groups increase or decrease simultaneously.

The Selbal algorithm models the outcome of interest (in our case the microbial composition of a specific seafloor type), and then identifies the smallest amount of taxa to put in a balance that gives a high predictive accuracy of the given seafloor. In short: Which taxa should be put in the balance so that the result is statistically different for samples from a given seafloor and the other seafloor types.

For example, looking at the Black and Yellow seafloors versus the rest (Figure 5E in the main manuscript), the selbal algorithm selected *Sulfurovaceae* against *Desulfurococcaceaea*. The formula is now:

$$\sqrt{\frac{(1)*(1)}{(1)+(1)}}*\log\frac{(counts\ Sulfurovaceae)^{1/1}}{(counts\ Desulfurococcaceaeae)^{1/1}}$$

The balance can now be calculated for all samples, and plotted in various ways. In figure 5, we decided to add depth on the y axis. Results for the samples belonging to the Black and Yellow patches are indeed very different from the others.

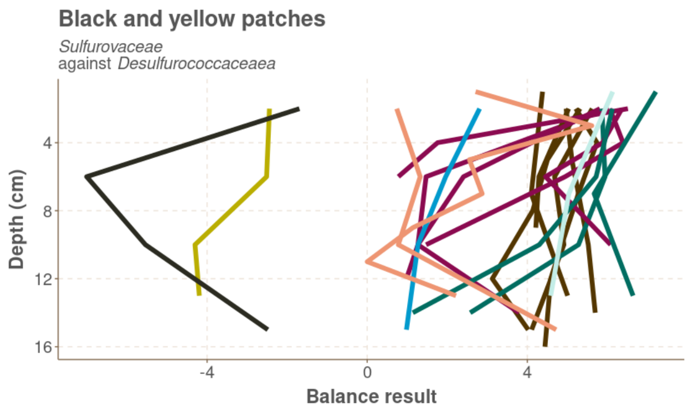

Quinn, T. P., Gordon-Rodriguez, E., and Erb, I. (2021). A Critique of Differential Abundance Analysis, and Advocacy for an Alternative. doi: 10.48550/arXiv.2104.07266.

Rivera-Pinto, J., Egozcue, J. J., Pawlowsky-Glahn, V., Paredes, R., Noguera-Julian, M., and Calle, M. L. (2018). Balances: a New Perspective for Microbiome Analysis. *mSystems* 3, e00053-18. doi: 10.1128/mSystems.00053-18.
